# Supplementary material for: Global and regional ecological boundaries explain abrupt spatial discontinuities in avian frugivory interactions
Source: Nat Commun. 2022 Nov 14;13:6943. doi: 10.1038/s41467-022-34355-w (PMC9663448; doi:10.1038/s41467-022-34355-w)
Supplement: Supplementary file 1 — Supplementary Information [file 41467_2022_34355_MOESM1_ESM.pdf]

Supplementary Information

Global and regional ecological boundaries explain abrupt spatial discontinuities in avian frugivory interactions

Lucas P. Martins*, Daniel B. Stouffer, Pedro G. Blendinger, Katrin Böhning-Gaese, Galo Buitrón-Jurado, Marta Correia, José Miguel Costa, D. Matthias Dehling, Camila I. Donatti, Carine Emer, Mauro Galetti, Ruben Heleno, Pedro Jordano, Ícaro Menezes, José Carlos Morante-Filho, Marcia C. Muñoz, Eike Lena Neuschulz, Marco Aurélio Pizo, Marta Quitián, Roman A. Ruggera, Francisco Saavedra, Vinicio Santillán, Virginia Sanz D'Angelo, Matthias Schleuning, Luís Pascoal da Silva, Fernanda Ribeiro da Silva, Sérgio Timóteo, Anna Traveset, Maximilian G. R. Vollstädt, Jason M. Tylianakis*

*Corresponding authors. Email: martinslucas.p@gmail.com (L.P.M); jason.tylianakis@canterbury.ac.nz (J.M.T)

Supplementary Methods

Standardizing the taxonomy

Considering the variety of authors and studies in our dataset, which identified plants and birds with differing resolution, it was necessary to reduce the taxonomic uncertainty in a uniform way. For this, we extracted the frugivore and plant species lists from all networks and performed a series of filters in order to remove non-existent species names (e.g., morphospecies labels) and standardize synonymous names according to reference databases.

Frugivore species

To account for spelling errors, we checked the matching of frugivore species names in our database to those from several taxonomic sources using the Global Names Resolver (GNR)[1]. We accessed this database using the function *gnr_resolve* from the R package taxize[2] (Supplementary Fig. 3; step 1). This function provides a matching score and the name from any of GNR's sources that most closely matches each name in our species list. Matching is determined by a combination of checking for exact matches against the names in the data sources and fuzzy matching (of canonical forms or parts of the names) using the TaxaMatch algorithm[3]. Because we were only interested in birds, we used the function *classification* from the same package to retrieve the taxonomic hierarchy and remove non-avian species, using the National Center for Biotechnology Information (NCBI)[4] as the reference database (Supplementary Fig. 3; step 2). For those species classified as birds, we used the function *gnr_resolve* one more time using BirdLife International[5] as the reference database (Supplementary Fig. 3; step 3). We used data from the Integrated Taxonomic Information System (ITIS)[6] and the *synonyms* function from the taxize package[2] to obtain the synonyms of the species cross-checked with BirdLife International, as well as of those that were not found in the BirdLife database but were previously classified as birds (Supplementary Fig. 3; step 4). We did this because, while obsolete bird species names usually did not have a match in BirdLife, one of its synonyms could: e.g., the black-fronted piping-guan was not found in the BirdLife database when its former scientific name, *Penelope jacutinga*, was entered; however, its currently accepted scientific name, *Pipile jacutinga*, was found as being one of the synonyms of *Penelope jacutinga*, and this synonym was revealed during step 4.

We also downloaded the Handbook of the Birds of the World (HBW) and BirdLife International (version 4.0)[7] and automatically checked for matches of species names in our frugivore list with the names from the columns 'scientific name' and 'synonym' of the HBW-BirdLife spreadsheet (Supplementary Fig. 3; step 5). By doing this, we were able to retrieve all the scientific names associated with the matched name in HBW-Birdlife. We used a fuzzy matching algorithm based on the Levenshtein distance between two strings to search for other possible names on the HBW-BirdLife spreadsheet for the species without good matches in any of the GNR's sources or BirdLife International, as well as for those species that were not found in the ITIS database (Supplementary Fig. 3; step 6). On some occasions, even this fuzzy matching algorithm could not find matches for a species name, which usually occurred when the genus name was incorrect or obsolete (note that in the vast majority of cases obsolete scientific names were fixed during steps 4 and 5, but some obsolete names were not present in either the ITIS or HBW-BirdLife databases). For those species, we automatically searched for their epithet names

in the columns ‘scientific name’ and ‘synonym’ of HBW-BirdLife and retrieved only those that had one single match in each column (Supplementary Fig. 3; step 7). The reason for restraining our search for those with one single match is because some epithet names are common and do not necessarily represent the same species. However, even this restriction is not a guarantee that the species with a given epithet in our list is the same species with the epithet in HBW-Birdlife, since a misspelled epithet name may coincidentally match the epithet of other species. Thus, we checked manually the taxonomy of all species corrected using this method (N = 17 species). We did this by searching for both the original species name (before the data cleaning process) and the matched name in the Avibase[8] and BirdLife[5] databases. By applying this series of filters, we were able to correct and validate the names and synonyms of 1,019 bird species. For the remaining species, we checked the taxonomy manually by inspecting the same databases as in the previous step.

Finally, we generated a list object in R[9] in which element names correspond to scientific names accepted by either BirdLife International - obtained using the *gnr_resolve* function from the taxize package[2] in 28/07/2020 - or HBW-BirdLife[7], while strings within elements correspond to all their synonyms and original species names. We used this list to standardize the taxonomy of the bird species in our local networks, so that synonyms would not be treated as different species (i.e., if two species were synonyms, they were attributed the same name in the local networks). All species that were removed during the cleaning process (non-bird species and those without genus and/or species names, such as Undefined sp. and *Turdus* sp.) were removed from our local networks and further analyses (N = 82 species). Around 86% of frugivore species remained per network after the data cleaning. Supplementary Figure 3 shows a summary of the steps of the frugivore data cleaning.

Plant species

We checked the matching of plant species names with several taxonomic sources from the Global Names Resolver (GNR)[1] using the function *gnr_resolve* from the taxize package[2] (Supplementary Fig. 4; step 1), as with birds above. For those species without matches in any of GNR’s sources, we applied a fuzzy matching algorithm based on the Levenshtein distance between two strings to compare these species’ names with the matched names from GNR (Supplementary Fig. 4; step 2). We did this because some of the species’ names without matches in our step 1 were misspelled names of plant species already included in our dataset but not found by the *gnr_resolve* function. After this process, we relied on the *gnr_resolve* function one more time to compare the list of matched names from GNR with the list from the International Plant Names Index (IPNI)[10] (Supplementary Fig. 4; step 3). The reason for using *gnr_resolve* twice is because we first wanted to make sure that the species had a match with at least one of the taxonomic sources from GNR (i.e., confirm that it is a scientific name) and then check whether the matched name represents a scientific name accepted by IPNI. By doing this, we were able to evaluate which species had high matching scores during our first step but not during the third, indicating that they are not internationally accepted scientific names.

We used data from the Tropicos database[11] to obtain the synonyms of the plant species that had been cross-checked with IPNI. We also relied on the iPlant Taxonomic Name Resolution Service[12] to complement the synonyms list and retrieve the most recent accepted names of the species (Supplementary Fig. 4; step 4). Using this series of filters, we were able to correct and validate the names and synonyms of 1,562 plant species. Finally, we generated a list

object in R[9] in which element names correspond to accepted scientific names of species (cross-checked with the IPNI database on 15/09/2020) and strings within elements correspond to all their synonyms and original species names (before the data cleaning process). We used this list to standardize the taxonomy of the plant species in our local networks, as we did for birds.

Because our plant list contained several cases in which two (or more) accepted species shared a synonym within their elements (*N* = 121), we had to deal with the standardization of these names. We did this by attributing the same name for all the occurrences of the species sharing a synonym only if the shared name was already present in our dataset. For example, *Cecropia digitata* is one of the synonyms of *C. angustifolia*, *C. obtusifolia* and *C. pachystachya* (and is therefore within the elements of these three species), but *C. digitata* was not present in any of the networks in our dataset, such that we could maintain the names C. *angustifolia*, *C. obtusifolia* and *C. pachystachya* in our local networks. We did this because shared synonyms that were not present in our dataset usually represented obsolete species that are no longer accepted. Alternatively, for the cases in which the shared synonym was present in our dataset (*N* = 37), we attributed the same name in the local networks for all the species that shared that given name. We adopted this conservative approach because, in this case, shared synonyms were usually species that were described multiple times by different authors, or species with several subspecies and varieties (note, however, that authors rarely include this level of taxonomic information on networks). Therefore, the shared name could potentially be any of the species that possess it as one of its synonyms.

Considering the high number of species (*N* = 184) with a valid genus name but without a valid epithet name (as indicated by the absence of matches in our steps 1 and 3, or by the low matching scores to any of the GNR's sources), as well as unresolved species names without good matches in the IPNI database (hereafter, *problematic species*) in our plant species list, we added two steps to evaluate whether such problematic species could be considered as a separate species from the other species in our dataset. For example, a species without an epithet (e.g., labelled in a study as '*Miconia* sp.') could still be treated as a distinct species in the analysis, provided we could be certain that it was not the same as another congeneric (*Miconia*) species, with or without epithet, in our dataset. Similarly, an unresolved species name that is not internationally accepted could only be considered as a distinct species in our analysis if we could disentangle it from its congeneric species in the dataset. Importantly, we did not perform these additional steps for birds because there were very few cases of birds with valid genus but invalid epithet names.

To determine whether problematic species could be treated as a distinct species for analysis, we evaluated whether the distribution of any of the congeners of problematic species in our dataset overlapped with the location of the problematic species, such that we cannot be confident that the problematic species is not simply another occurrence of one or more of its congeners already in the dataset. For this process, we used the coordinates of the networks in which each problematic species occurred and generated buffer zones (diameter = 500 km) around these network locations. Considering that the size of the buffer zones could potentially affect our results, we also conducted the analysis using buffer zone sizes of 100 km and 1000 km (note, however, that our results still hold independently of the buffer zone size used; see Supplementary Tables 9-32). We collected occurrence data for all other species in the same genus in our dataset to evaluate whether the occurrence points of any of these congeneric species overlapped with the buffer zone of the problematic species (Supplementary Fig. 4; step 5). For collecting occurrence points, we used data from the Global Biodiversity Information Facility (GBIF)[13] and applied a series of filters (for details, see the Occurrence data section below). If the

occurrence points of at least one of the congeneric species overlapped with the buffer zone of a given problematic species, we assumed that this problematic species could not be considered, with confidence, as a unique species in our dataset. Conversely, if none of the occurrence points of congeneric species overlapped with the buffer zone of the problematic species, we treated this problematic species as a separate species (Supplementary Fig. 5), provided that there were no other problematic species (without valid epithets) in the same genus from other studies in the dataset.

Alternatively, if a genus contained more than one problematic species in the same study (e.g., *Miconia* sp.1, *Miconia* sp.2), we assumed that the authors distinguished the congeners within the study. For the cases in which a problematic species occurred in a single study and was the only species belonging to that genus in our dataset, the original name of the species was maintained in the local network. However, if there were problematic species from the same genus in different studies, we needed to ascertain whether they could potentially be the same species. Our approach for dealing with this issue was to determine all the possible species that a problematic species could be in each location, and then compare the lists of possible species in each location to identify any overlap. To do this, we first generated buffer zones (as in step 5) for each network location in which these problematic species occurred and obtained occurrence data from GBIF for all known species belonging to that genus (see the Occurrence data section). We then checked whether there were congeneric species with occurrence points within the buffer zones of two (or more) problematic species belonging to the same genus (Supplementary Fig. 4; step 6). If yes, we could not consider that these problematic species were different from each other. Rather, in this case there was a chance that the problematic species were the same species whose occurrence points overlapped the buffer zones of both network locations (Supplementary Fig. 6). On the other hand, if there were no species whose distribution overlapped the buffer zones of both network locations, these problematic species could be considered as being distinct species in the dataset.

All species that were removed during the data cleaning process (i.e., the problematic species without a valid genus name, such as Rubiaceae sp. or Undefined sp.) were also removed from our local networks and further analyses (N = 166 species). Problematic species that could not be disentangled from resolved species or other problematic species in the dataset were named according to three distinct scenarios (for details, see the Alternative scenarios section). Around 89% percent of plant species remained per network after the data cleaning (note, however, that this percentage varies slightly depending on the scenario employed). Supplementary Figure 4 shows a summary of the steps of the plant data cleaning.

Occurrence data

We retrieved occurrence data from the Global Biodiversity Information Facility (GBIF)[13] using the function *occ_search* from the R package rgbif[14]. For each species, we only requested occurrence data for observations for which coordinate points were available and no geospatial issues were detected, as determined by GBIF's record interpretation. We also followed a previous study[15] and removed occurrence points with: (i) a coordinate uncertainty larger than 100 km (the size of our smallest buffer zone); (ii) those for which the collection date was before 1945, as older occurrence points are usually not properly geo-referenced[16]; (iii) those in which the number of counts associated with the occurrence point was zero; and (iv) those in which the 'basis of record' was not an observation or a preserved specimen.

In addition, we used the function *clean_coordinates* from the R package CoordinateCleaner[17] and land mass and country data (with a 1:10m scale) from Natural Earth[18] to remove occurrence points for which the coordinates: (v) fell within the ocean or outside the borders of the country where they were recorded, both of which indicate data-entry errors, (vi) were located around the country capital or the centroid of the country, indicating imprecise geo-referencing based on inadequate sampling site descriptions, (vii) both latitude and longitude were zero or had equal values, indicating failed geo-referencing, and (viii) were located around a biodiversity institution, suggesting that records might represent specimens that were erroneously geo-referenced to museums, herbaria or universities instead of their sampling localities[17]. After applying this series of filters, 456,582 occurrence points were retrieved for 610 plant species in our dataset. These occurrence points were used for disentangling ‘problematic’ species during step 5 of the plant species cleaning process (Supplementary Fig. 5).

Because the next step required us to retrieve occurrence data for all known species belonging to a given genus, we used the function *name_lookup* from the R package rgbif[14] to search for all accepted species names associated with the genus name. We used the same set of filters previously described to obtain the occurrence points for each species during the step 6 of the plant species cleaning process (Supplementary Fig. 6). In the end, 994,270 occurrence points were retrieved for 4,793 plant species.

Alternative scenarios

We used three distinct scenarios for attributing names for problematic plant species that could not be considered as unique species in our dataset. In the first scenario, we removed from the local network any problematic species whose buffer zone was overlapped by the distribution of ‘resolved’ congeneric species in the dataset (step 5 of the plant species cleaning process). For example, if the buffer zone of the problematic species ‘*Miconia* sp.’ was overlapped by other resolved *Miconia* species in the dataset, we removed the species *Miconia* sp. (and all of its interactions) from its local network. We adopted this strategy rather than considering that the problematic species and the resolved species that overlap its buffer zone are the same because such problematic species could potentially be any of the resolved species that overlap its buffer zone. This, in turn, made it impractical to attribute the name of the resolved species to the problematic species in cases where the buffer zone of the problematic species was overlapped by several resolved species. In addition, our first scenario considers all problematic species that could not be disentangled from each other (step 6 of the plant species cleaning process) as being the same species. For example, if two problematic species labelled as ‘*Coussapoa* sp.’ in two separate local networks could not be disentangled because there are congeneric species simultaneously overlapping the buffer zones of both network locations (Supplementary Fig. 6), we attributed the same name to these two problematic species.

Alternatively, our second scenario treats problematic species as being unique. Therefore, a unique name was given for the problematic species whose buffer zone was overlapped by ‘resolved’ congeneric species in the dataset. For instance, the problematic species ‘*Miconia* sp.’ from the example above would receive a unique name in the second scenario instead of being removed from its local network. In this scenario, we also attributed unique names for problematic species that could not be disentangled from each other. For example, each of the two problematic *Coussapoa* species mentioned above would receive a unique name instead of sharing the same name.

Finally, the third scenario removes from the local networks all plant species that could not be considered as being unique species in the dataset and is therefore our most conservative scenario (which was used for obtaining the results presented in the main text). Because these three different scenarios could affect our response variables, we repeated the analyses using the sets of networks from all scenarios. Notably, results remained qualitatively the same independently of the scenario used in the analyses (Supplementary Tables 9-32).

Sensitivity analysis

Considering that the threshold of minimum network size used for analyses is arbitrary and has the potential to affect the reported patterns, we performed a sensitivity analysis to evaluate how sequentially removing local networks based on their size would affect the estimates (t and F values) and significance (obtained using a combination of Generalized Additive Models and Multiple Regression on distance Matrices) of our predictor variables. We did this by sequentially removing all networks below a specified threshold of size (i.e., class of network size) in our dataset, from smallest to largest (Supplementary Fig. 22). Note, however, that although we had 60 different classes of network sizes (minimum network size = 8 species; maximum network size = 238 species), we were only able to perform the analysis up to the removal of networks with 71 (or fewer) species (which represented 183 out of the 196 local networks in our dataset). We could not remove the local networks with larger sizes (N = 13 networks) because removing any of these remaining networks would lead to our Generalized Additive Models (GAMs) having more coefficients than data.

We found that removing small networks from our dataset did not strongly affect our results; for instance, removing networks with up to 10 species (which represent 17 out of 196 local networks in our dataset) would not affect any of the reported patterns (Supplementary Figs. 23 and 24). We also highlight that even though all estimates tend to approach zero with the removal of larger networks, this is partially because beyond a certain number of network removals there are too few data points and insufficient range of the predictor value for the model to be able to detect an effect.

Importantly, of the significant effects in the full model using interaction dissimilarity as response, only biome boundaries seem to be sensitive to the sequential removal of small networks from the dataset (Supplementary Fig. 23). However, biome boundaries explained a relatively low unique proportion of the variation in interaction dissimilarity in our full model. In fact, most of the deviance explained by biomes was shared with ecoregions (Supplementary Fig. 12), which is likely because biomes share boundaries with ecoregions and the latter explain finer-resolution environmental differences[19]. This strong effect of ecoregion borders on interaction dissimilarity is corroborated in our sensitivity analysis: the effect of ecoregions remained significant even after the removal of networks with up to 57 species (which represented around 88% of the local networks in our dataset; Supplementary Fig. 22).

In addition, the two significant effects in our full model using network dissimilarity as response variable were very robust to the removal of small networks from the dataset (Supplementary Fig. 24). More specifically, the effect of spatial distance remained significant even after the removal of networks with up to 22 species (which represented around 50% of our local networks), while the effect of sampling intensity was still significant after the removal of networks with up to 32 species (~ 68% of the local networks in the dataset).

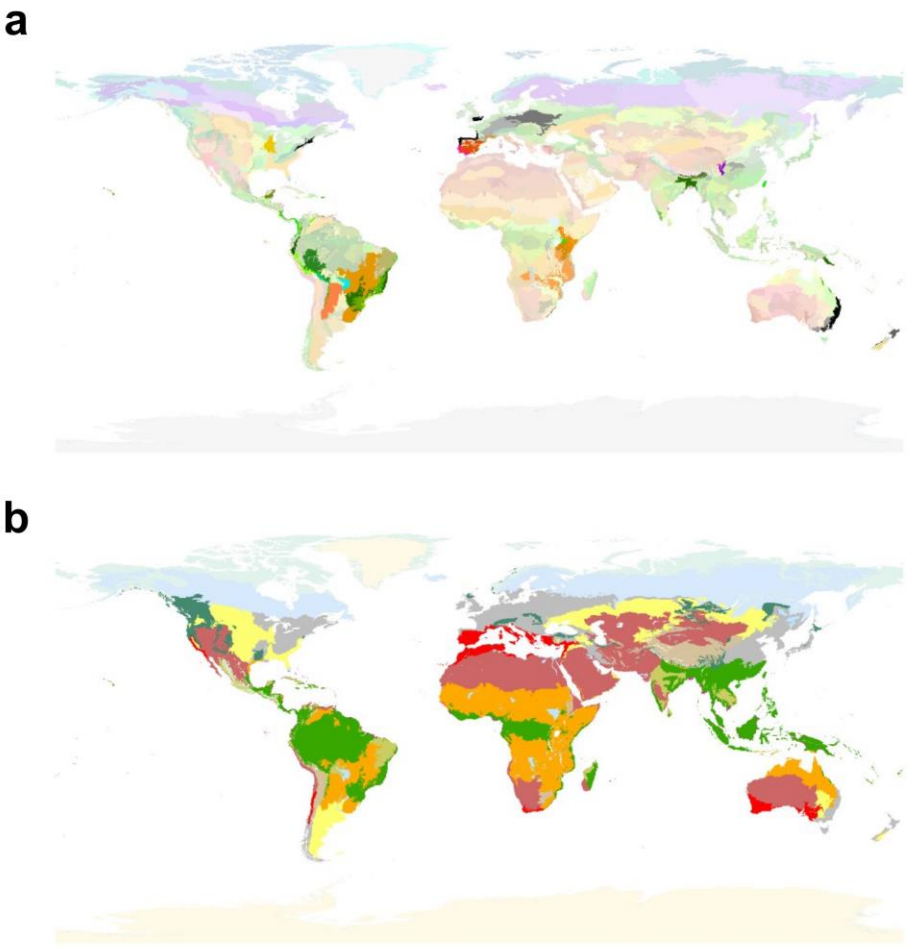

Supplementary Fig 1. Maps of ecoregions and biomes of the world. a Terrestrial ecoregions, with stronger color tones indicating the 67 ecoregions (out of 846) represented in our dataset. **b** Global biomes, with stronger color tones indicating the 11 biomes (out of 14) represented in our dataset. Ecoregions and biomes were defined based on the map developed by Dinerstein et al.[19] (available at https://ecoregions.appspot.com/ under a CC-BY 4.0 license).

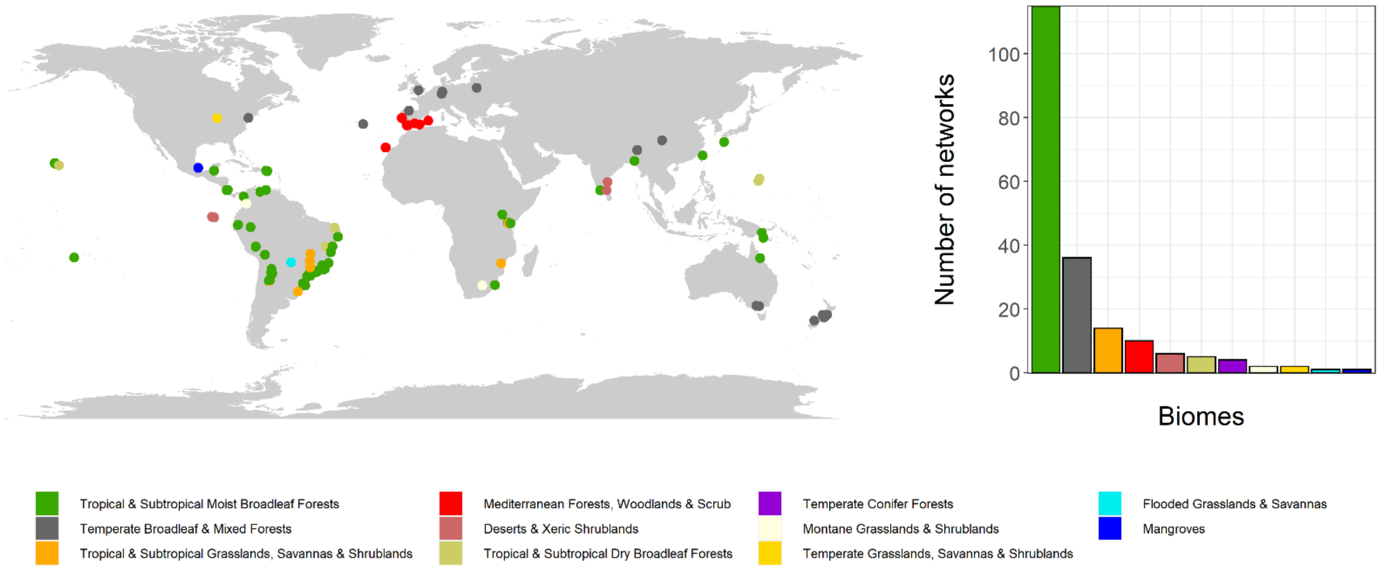

Supplementary Fig. 2. Geographic distribution of the 196 avian frugivory networks in our dataset. Local networks were distributed across 11 biomes, with most of these being located within a single biome, the Tropical & Subtropical Moist Broadleaf Forests, which covers around 11% of the world's ice-free land surface. Biomes were defined based on the map developed by Dinerstein et al.[19] (available at https://ecoregions.appspot.com/ under a CC-BY 4.0 license).

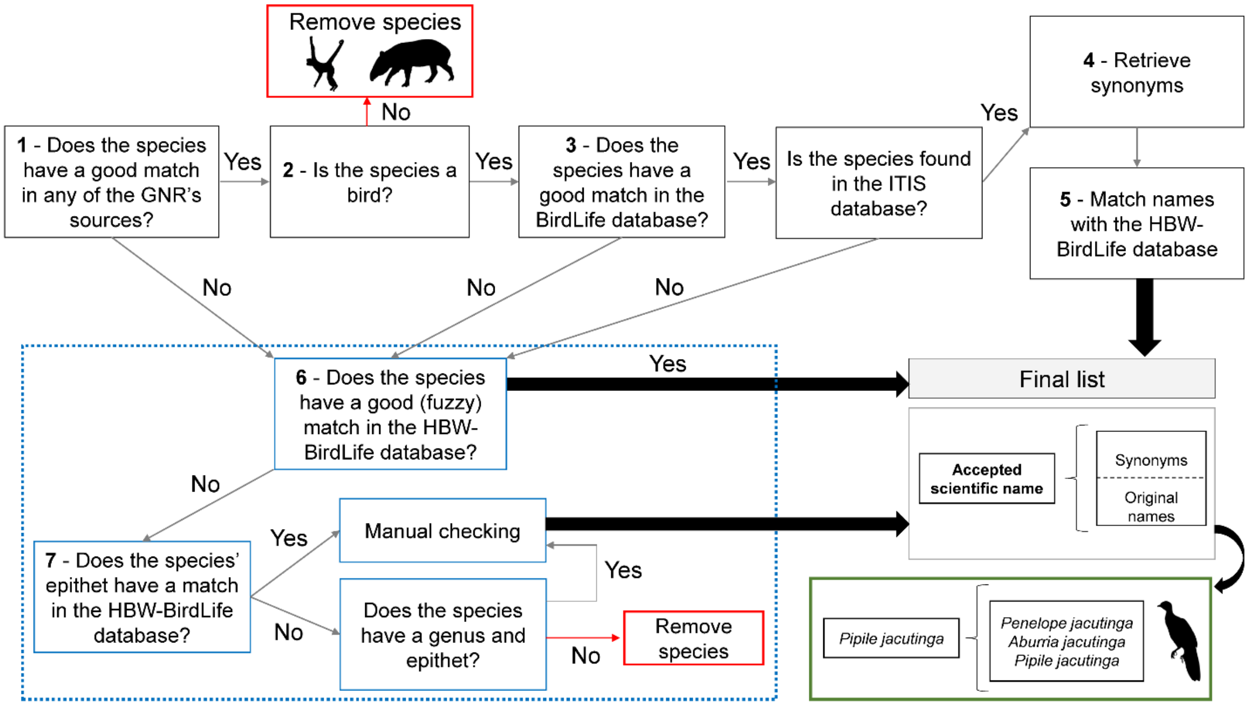

Supplementary Fig. 3. An overview of the steps for cleaning and standardizing the frugivore species data. Red boxes represent species that were removed from the analyses (non-avian species and species without epithet or genus names). The dashed box comprises the steps performed for the species without good matches in any of the Global Names Resolver (GNR) sources and in the BirdLife International database, or that were not found in the Integrated Taxonomic Information System (ITIS). The final list comprises elements whose names represent scientific names accepted either by the BirdLife International or by the Handbook of the Birds of the World and BirdLife International, and strings within elements comprise their synonymous and original names (before the cleaning process). For example, *Pipile jacutinga* (Cracidae) is the current accepted name of the black-fronted piping-guan, while its synonymous names include *Penelope jacutinga* and *Aburria jacutinga* (green box). All names (strings of synonyms) within elements (accepted names) were replaced by the element name in the local networks, such that a given species had the same name for all its occurrences in the entire database. Numbers inside boxes correspond to the steps of the frugivore data cleaning process. Silhouettes were obtained from http://phylopic.org under a Public Domain license.

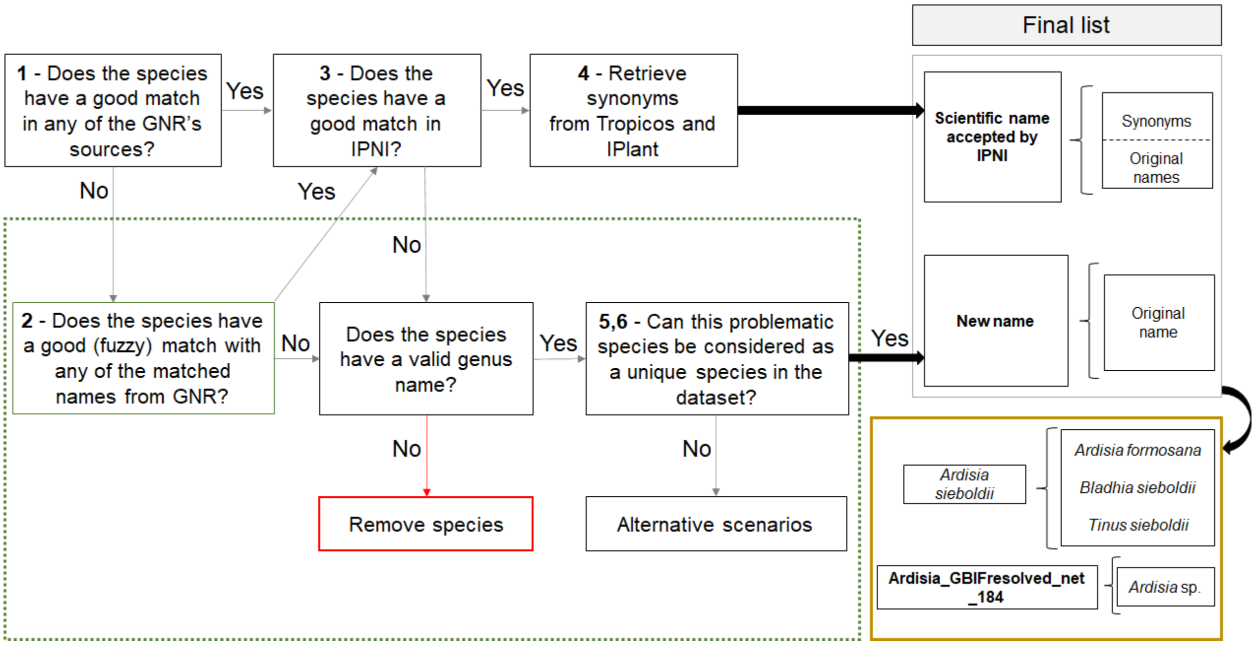

Supplementary Fig. 4. Overview of the steps for cleaning and standardizing the plant species data. The red box represents species that were removed from the analyses (species without valid genus names). The dashed box comprises the steps performed for the species without good matches in any of the Global Names Resolver (GNR) sources and in the International Plant Names Index (IPNI) database (i.e., ‘problematic species’). We performed two steps to determine if problematic species could be considered as being unique species in our dataset (see steps 5 and 6 of the plant species data cleaning process described in the text and visualized in Supplementary Figs. 5 and 6). The final list comprises elements whose names represent scientific names cross-checked with the IPNI database and strings within elements comprise their synonymous and original names (before the cleaning process), or elements whose names represent new names given for problematic species that can be considered as unique species in our dataset, and the strings within elements comprise their original name. For example (yellow box), *Ardisia sieboldii* (Primulaceae) is a scientific name accepted by IPNI, while *A. formosana*, *Bladhia sieboldii* and *Tinus sieboldii* represent some of its synonymous names. Meanwhile, Ardisia_GBIFresolved_net_184 is the new name given for the problematic (but unique) species *Ardisia* sp., as revealed by the step 5 of the plant species data cleaning process. Note that, in the former case, all names (strings) within elements were replaced by the element name in the local networks, while in the latter case strings within elements were replaced by the element name only in the network where the problematic species was observed (in this example, network 184). Numbers inside boxes correspond to the steps of the plant data cleaning process. See the Alternative scenarios section for details on how we attributed names for plant species that could not be considered as unique species in our dataset.

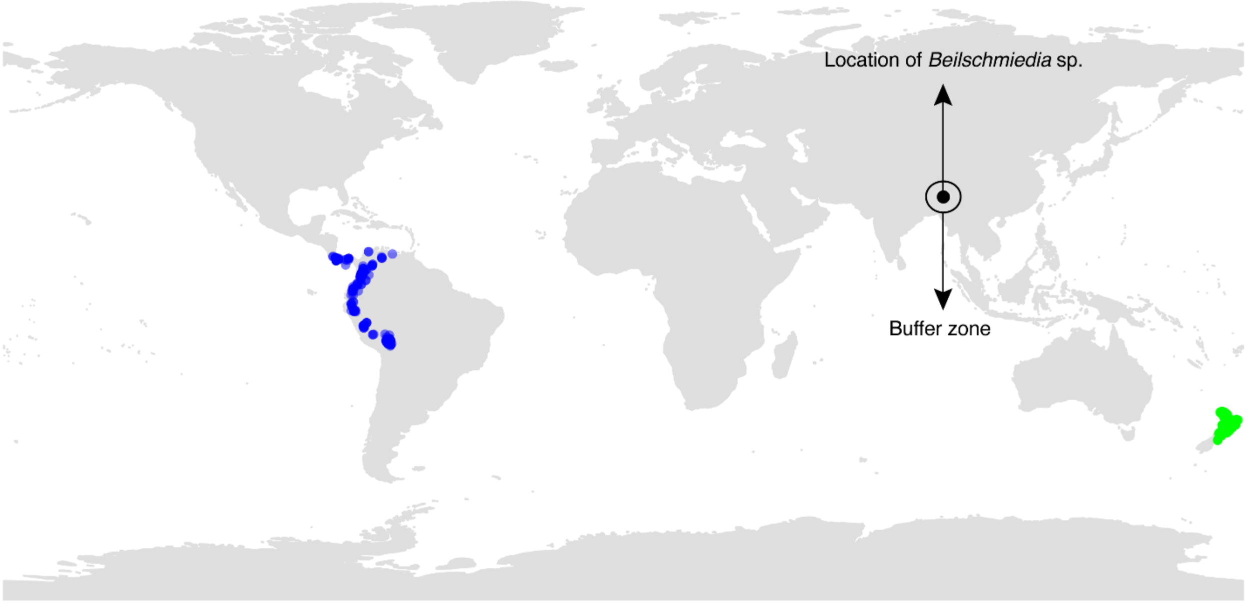

Supplementary Fig. 5. Graphical example (for an unresolved *Beilschmiedia* species) of step 5 of the plant species cleaning process. Coloured points indicate the distribution of *Beilschmiedia* species already contained within our dataset, and these are compared with the occurrence location of a 'problematic species' (a species with genus name only). The distributions of both *Beilschmiedia tawa* (green dots) and *Beilschmiedia tovarensis* (blue dots) do not overlap with the buffer zone of the problematic species *Beilschmiedia* sp., such that *Beilschmiedia* sp. can be considered as a separate species in our dataset.

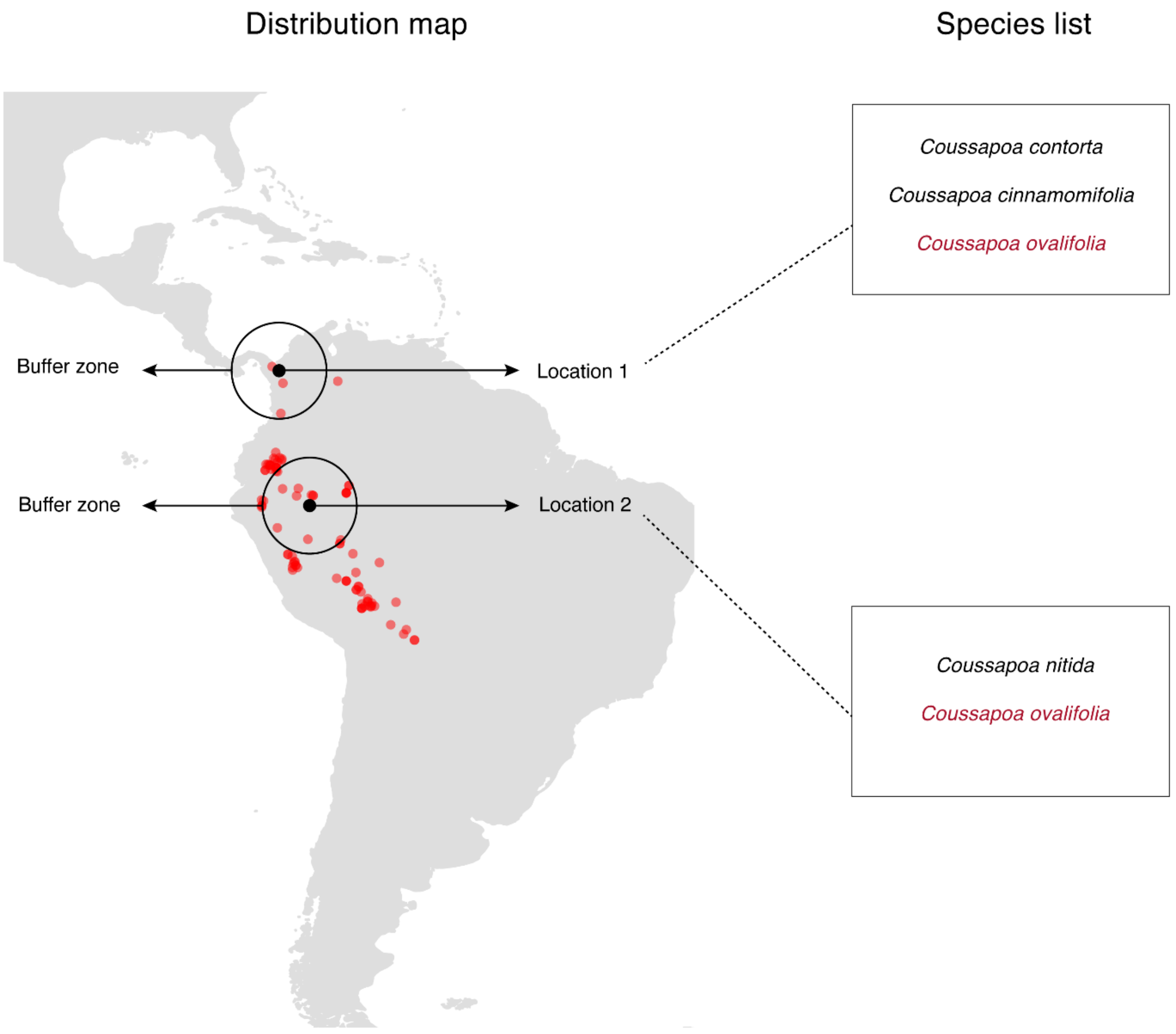

Supplementary Fig. 6. Graphical example of step 6 of the plant species cleaning process. In this example, there are two occurrences of species labelled '*Coussapoa* sp.' in separate studies (locations 1 and 2). The distribution of *Coussapoa ovalifolia* (red dots) simultaneously overlaps the buffer zones of two 'problematic species' (i.e., species with genus name only) belonging to the same genus, such that these problematic species could not confidently be considered as being separate species. A distribution map like this was created for all congeneric species with occurrence data in either buffer zone. Note that *C. ovalifolia* is present in the potential list of *Coussapoa* species in both network sites (other *Coussapoa* species were omitted in the species lists for clarity).

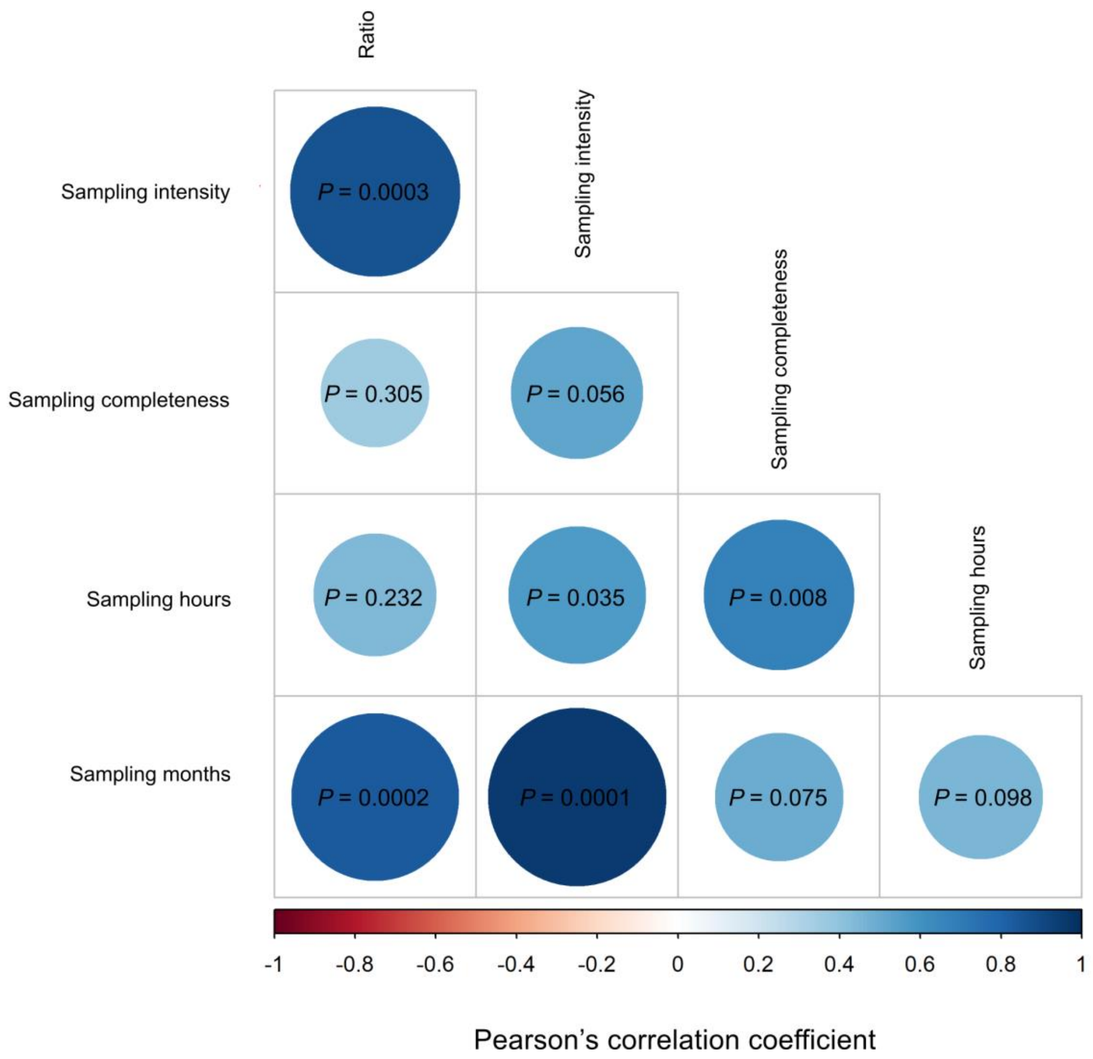

Supplementary Fig. 7. Results from Pearson's correlation tests between sampling metrics. For this analysis, we used the subset of networks sampled in Aotearoa New Zealand (N = 14). Numbers inside circles indicate P values obtained using a two-tailed Pearson correlation test. Sizes of the circles and colors are proportional to the correlation coefficient.

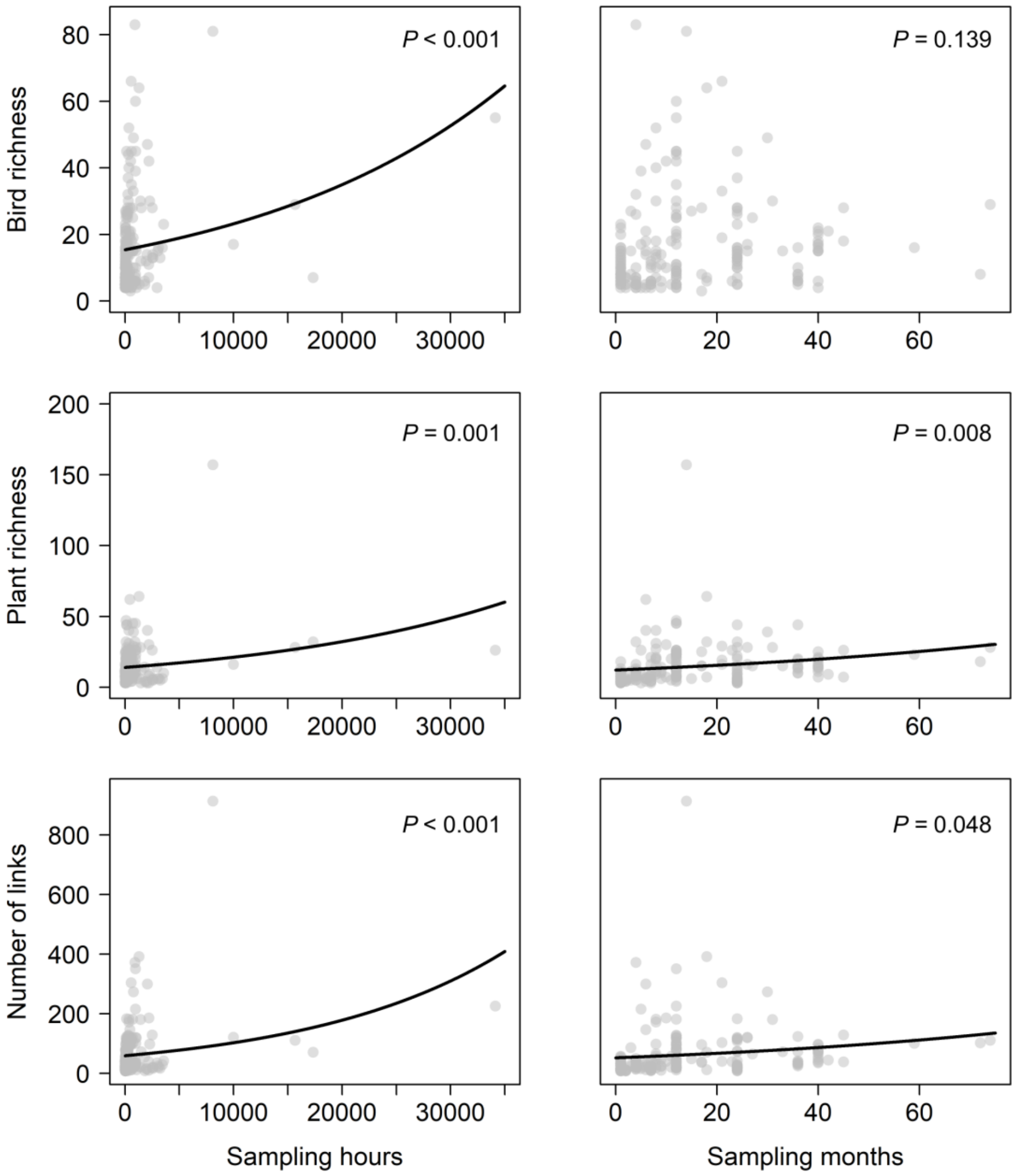

Supplementary Fig. 8. Relationships between network metrics and sampling hours and months. We used Generalized Linear Models (with Poisson errors, fitted with quasi-likelihood to deal with overdispersion) to obtain significance values. Points represent the 196 local frugivory networks in our dataset. Solid lines represent significant relationships.

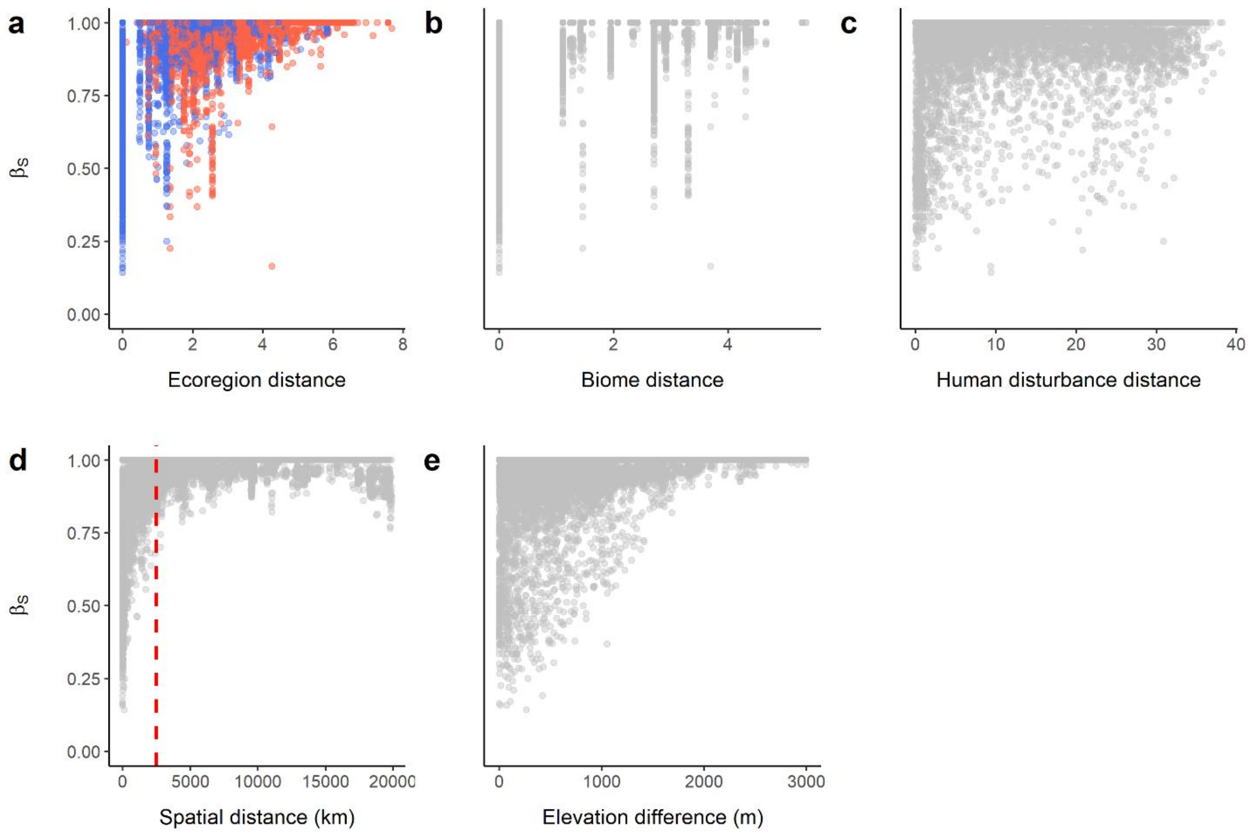

Supplementary Fig. 9. Scatterplots of the relationships between our predictor variables of interest (not those used for controlling sampling effects) and species turnover (β_S). a The relationship between the quantitative version (environmental dissimilarity) of ecoregion distance and species turnover; point colors indicate whether the pair of local networks belong to the same (blue) or distinct (red) biomes. **b** The relationship between the quantitative version (environmental dissimilarity) of biome distance and species turnover. **c** The relationship between local human disturbance distance and species turnover. **d** The relationship between spatial distance and species turnover. Note that, contrary to species interactions (Fig. 6 in the main text and Supplementary Fig. 14c), several networks still shared species beyond the threshold distance of 2,500 km (dotted red line). **e** The relationship between elevation difference and species turnover.

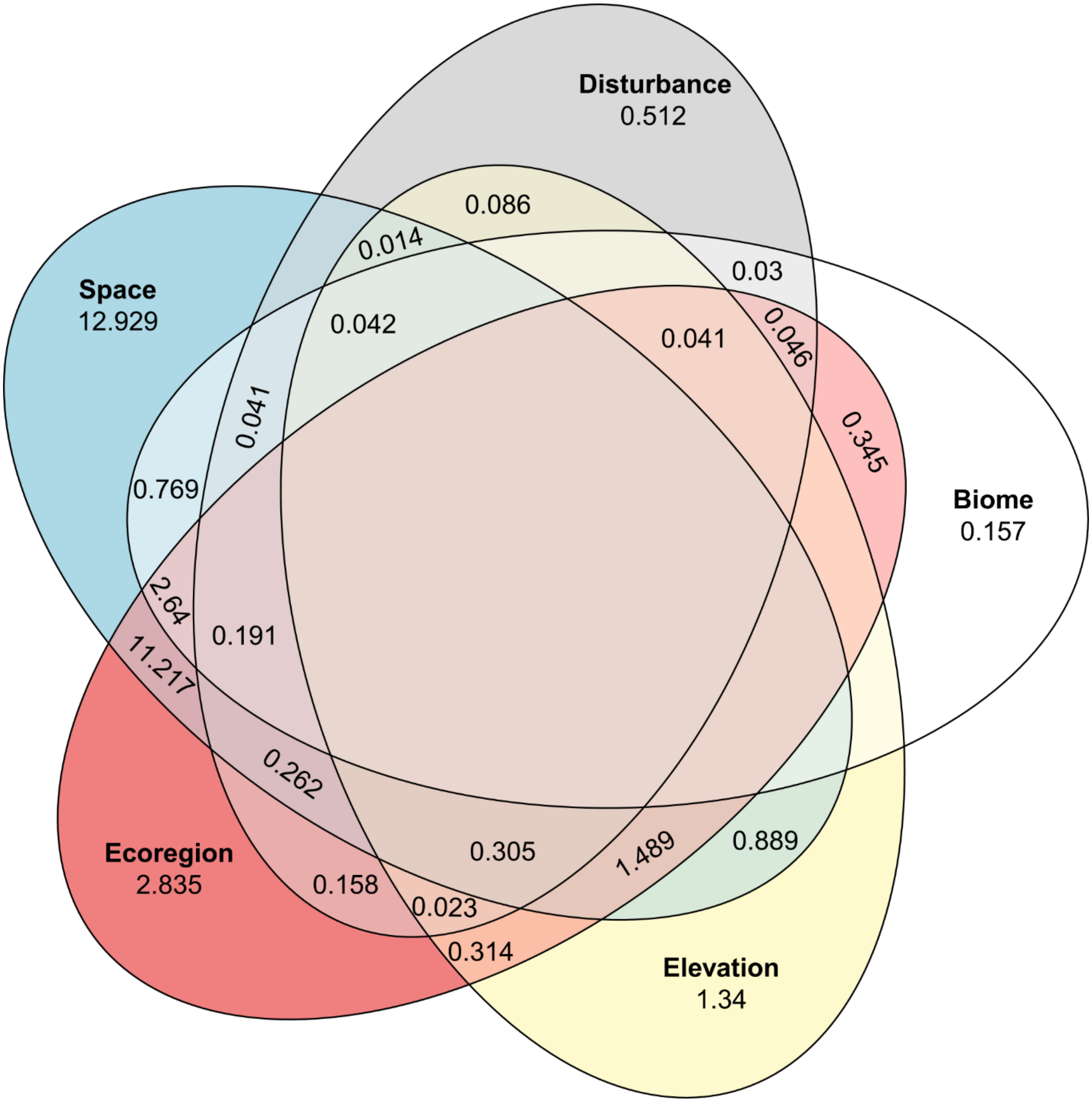

Supplementary Fig. 10. Venn diagram showing the relative contributions (%) of our main predictor variables to explaining the variation in species turnover (βs) across networks, calculated using deviance partitioning. Spatial distance alone explained the greatest proportion (12.9%) of the variation in species turnover, followed by the shared effect of spatial distance and ecoregion boundaries. Note that, to aid visualization, we only included our predictor variables of interest (i.e., not those used for controlling sampling effects). Terms that reduce explanatory power are not shown.

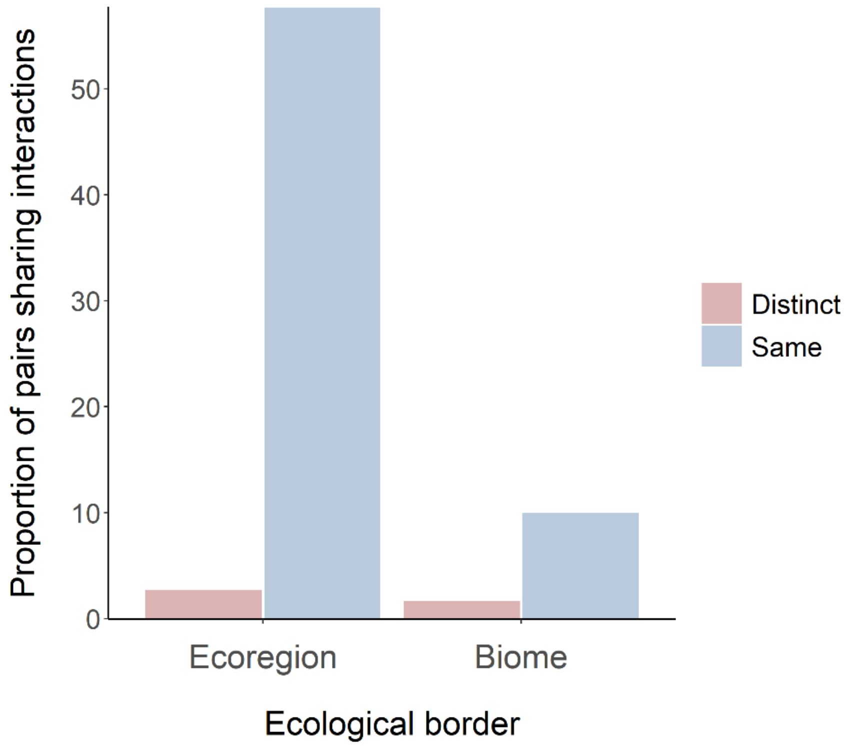

Supplementary Fig. 11. The effect of large-scale ecological boundaries on the proportion of pairs of local networks sharing interactions. Avian frugivory networks located within the same ecoregion/biome were more likely to share interactions than those located across distinct ecoregions/biomes. Note that over 50% of the pairs of networks located within the same ecoregion shared interactions.

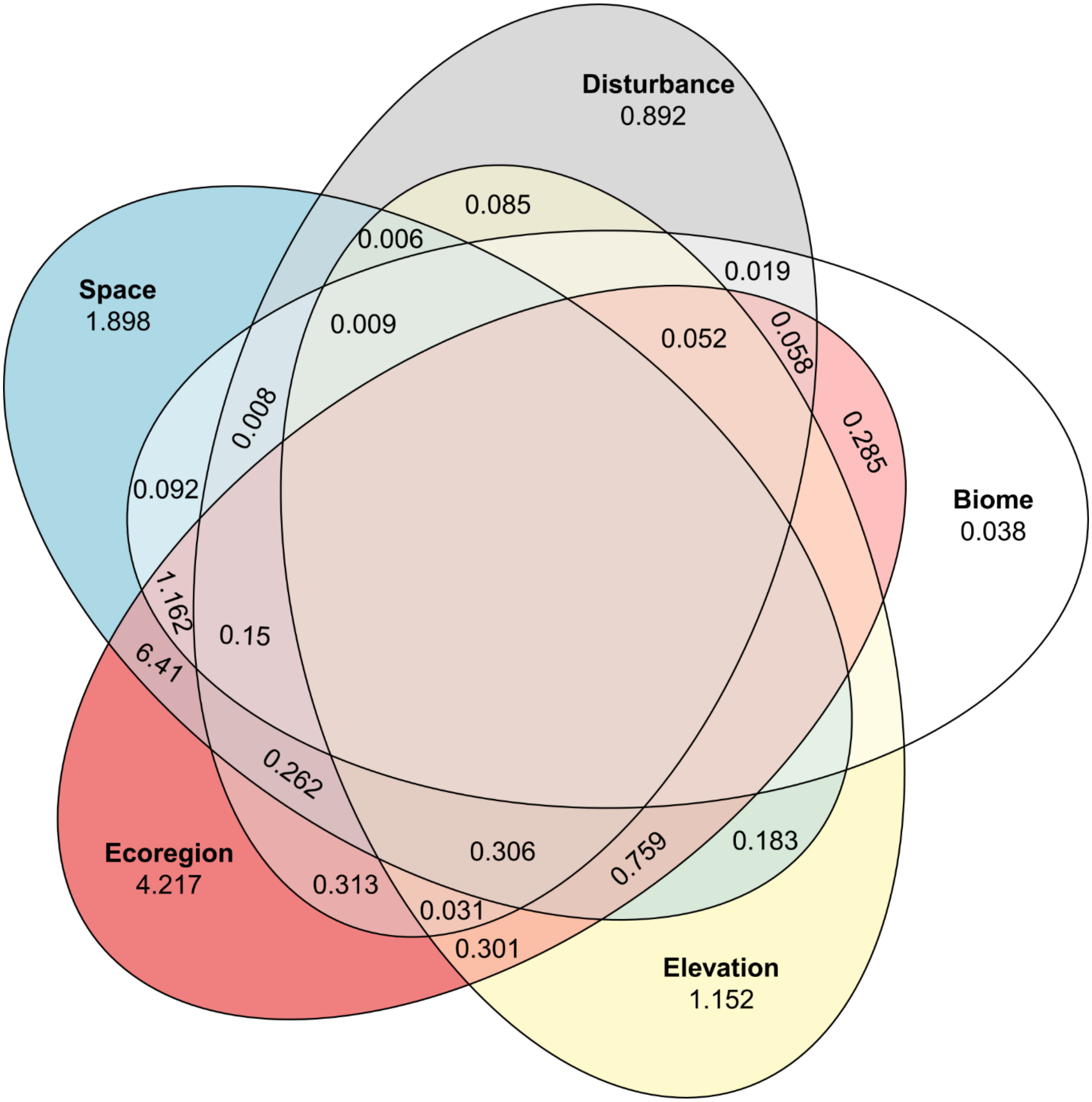

Supplementary Fig. 12. Venn diagram showing the relative contributions (%) of our main predictor variables to explaining the variation in plant-frugivore interaction dissimilarity (β_{WN}), calculated using deviance partitioning. The shared effect of ecoregions and spatial distance explained the greatest proportion (6.41%) of the variation in interaction dissimilarity, followed by the unique contributions of these two variables. Note that, to aid visualization, we only included our predictor variables of interest (i.e., not those used for controlling sampling effects). Terms that reduce explanatory power are not shown.

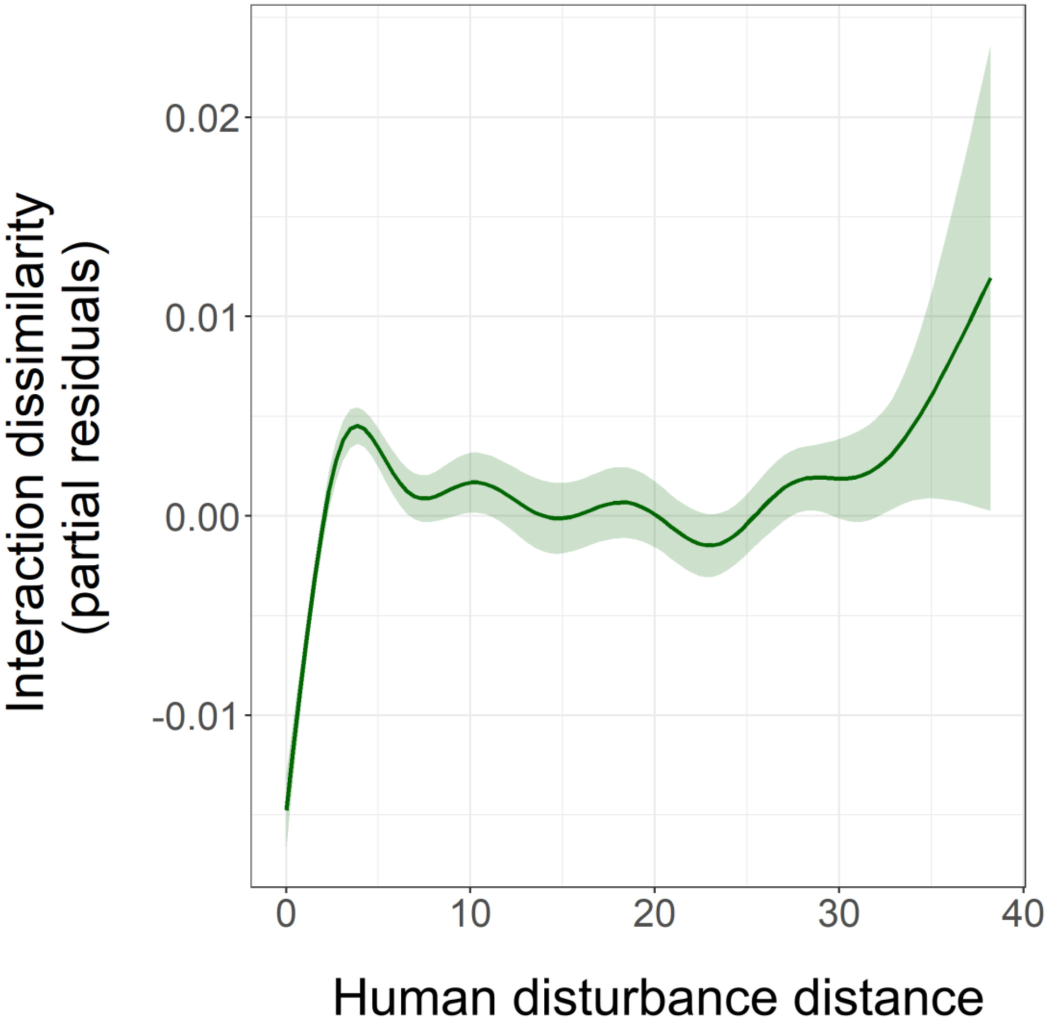

Supplementary Fig. 13. Partial effects plot of the relationship between human disturbance distance and interaction dissimilarity (β_{WN}). The smoothed line was fitted using a Generalized Additive Model (GAM) with interaction dissimilarity as response variable and all of our predictor variables included (see Table 1 in the main text). The lighter green area represents 2 standard errors above and below the estimate of the smooth being plotted.

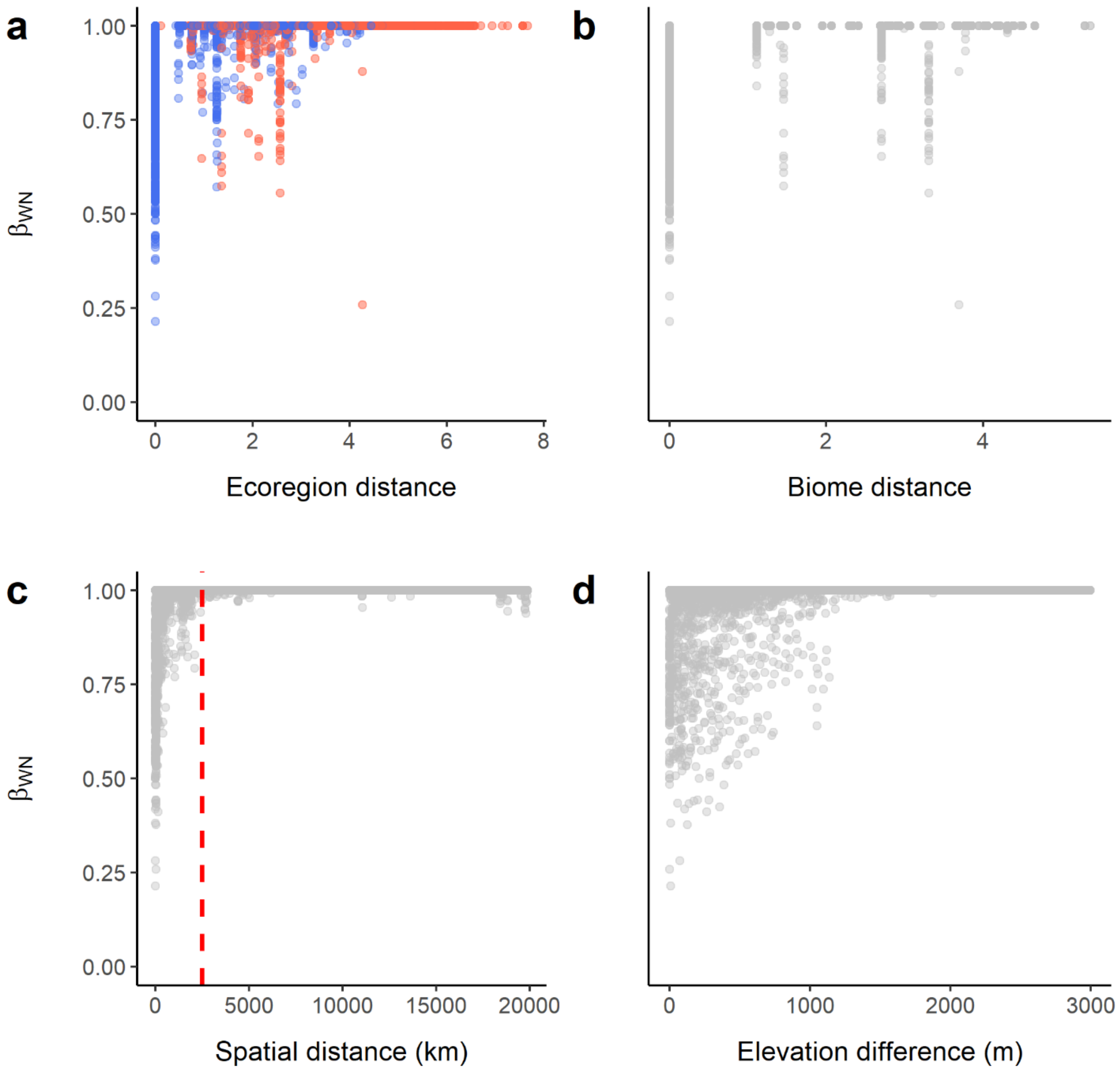

Supplementary Fig. 14. Scatterplots of the relationships between our predictor variables of interest (except human disturbance distance, which is presented in the main text) and interaction dissimilarity (β_{WN}). a The relationship between the quantitative version (environmental dissimilarity) of ecoregion distance and interaction dissimilarity; point colors indicate whether the pair of networks belong to the same (blue) or distinct (red) biomes. **b** The relationship between the quantitative version (environmental dissimilarity) of biome distance and interaction dissimilarity. **c** The relationship between spatial distance and interaction dissimilarity. Note that interaction dissimilarity increases sharply until a threshold distance of 2,500 km (dotted red line), beyond which few networks shared interactions (a similar pattern can be seen in Fig. 6 in the main text). **d** The relationship between elevation difference and interaction dissimilarity.

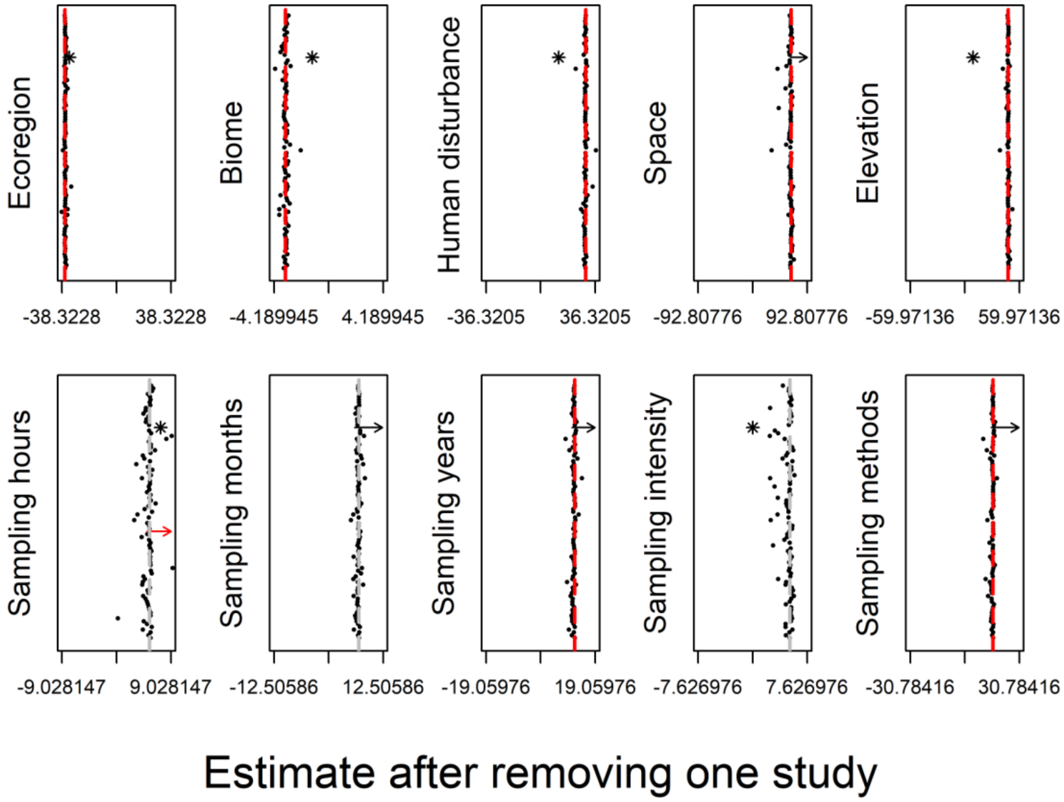

Supplementary Fig. 15. Effect of individual studies on estimates of t (for ecoregion and biome) and F values (for the remaining predictor variables) of Generalized Additive Models with interaction dissimilarity (β_{WN}) as response variable. Points represent estimate values after removing one study from the data, while asterisks indicate the estimates when the study with the greatest number of networks (N = 35) in our dataset (study ID 76)[20] is removed from the data. The estimates of the full model (with all studies included) are represented by the vertical lines. Red lines indicate a significant effect ($P < 0.05$), while gray lines indicate a non-significant effect. P values were calculated using a two-tailed statistical test that combines Generalized Additive Models (GAM) and Multiple Regression on distance Matrices (MRM). In this approach, the non-independence of distances from each local network is accounted for in the hypothesis testing by performing 1,000 permutations of the response matrix (see Methods). The range of the x-axis was defined as ± 3 times the standard deviation of the estimates. Arrows indicate outliers beyond this range (black: when study 76 is removed; red: when other studies are removed).

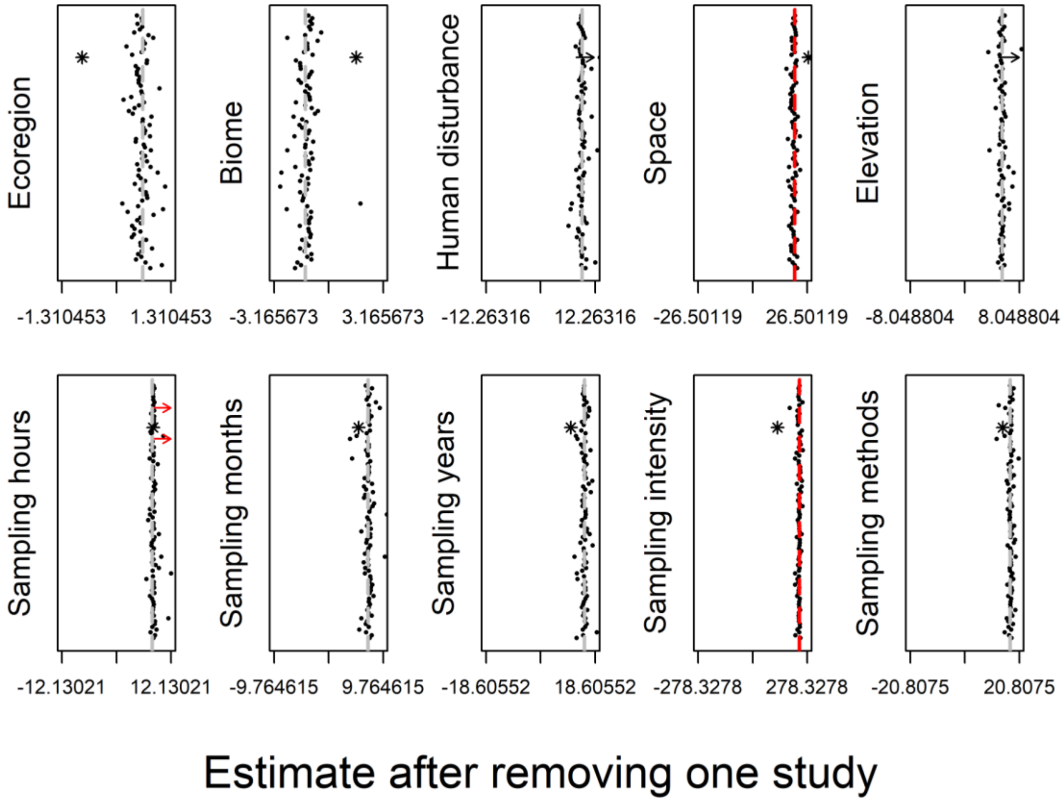

Supplementary Fig. 16. Effect of individual studies on estimates of t (for ecoregion and biome) and F values (for the remaining predictor variables) of Generalized Additive Models with network structural dissimilarity as response variable. Points represent estimate values after removing one study from the data, while asterisks indicate the estimates when the study with the greatest number of networks (N = 35) in our dataset (study ID 76)[20], is removed from the data. The estimates of the full model (with all studies included) are represented by the vertical lines. Red lines indicate a significant effect ($P < 0.05$), while gray lines indicate a non-significant effect. P values were calculated using a two-tailed statistical test that combines Generalized Additive Models (GAM) and Multiple Regression on distance Matrices (MRM). In this approach, the non-independence of distances from each local network is accounted for in the hypothesis testing by performing 1,000 permutations of the response matrix (see Methods). The range of the x-axis was defined as ± 3 times the standard deviation of the estimates. Arrows indicate outliers beyond this range (black: when study 76 is removed; red: when other studies are removed).

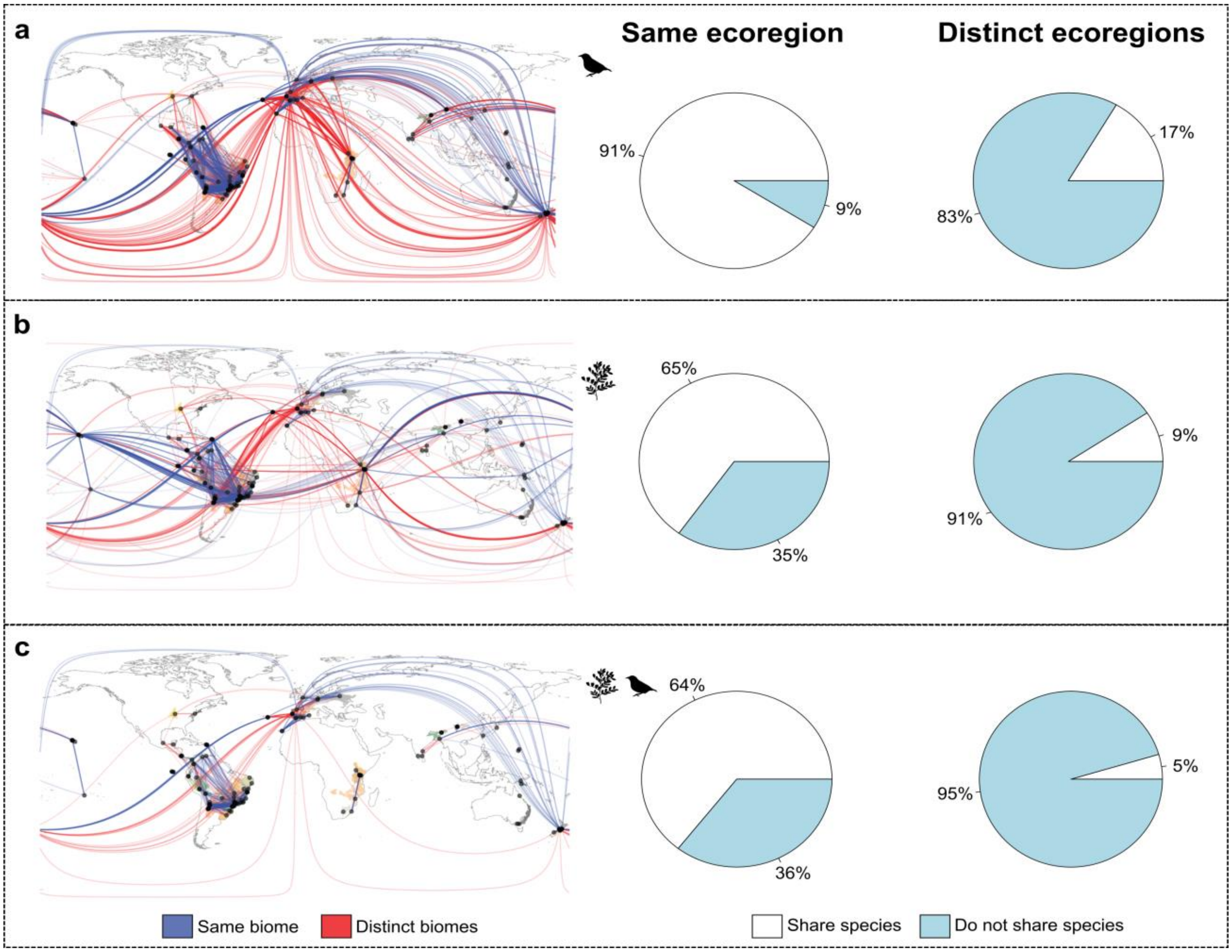

Supplementary Fig. 17. Plant and bird species connecting local networks, ecoregions and biomes. World map with points representing the 196 local avian frugivory networks in our dataset. As in Fig. 2 in the main text, colors of shaded areas represent the 67 ecoregions where networks were located, with similar colors indicating ecoregions that belong to the same biome. Lines represent the connections (shared species) plotted along the great circle distance between networks. Blue lines represent connections within biomes, while red lines represent connections across biomes. Stronger color tones of lines indicate higher similarity of species ($1-\beta_S$) between networks. **a** Lines represent connections between networks sharing bird species. Pie charts depict the proportion of pairs of local networks sharing bird species across vs. within ecoregions. **b** Lines represent connections between networks sharing plant species. Pie charts depict the proportion of pairs of local networks sharing plant species across vs. within ecoregions. **c** Lines represent connections between networks sharing both plant and bird species. Pie charts depict the proportion of pairs of local networks sharing both plant and bird species across vs. within ecoregions (see Fig. 2 for the world map of shared plant-frugivore interactions). Ecoregions and biomes were defined based on the map developed by Dinerstein et al.[19] (available at https://ecoregions.appspot.com/ under a CC-BY 4.0 license). Silhouettes were obtained from http://phylopic.org under a Public Domain license.

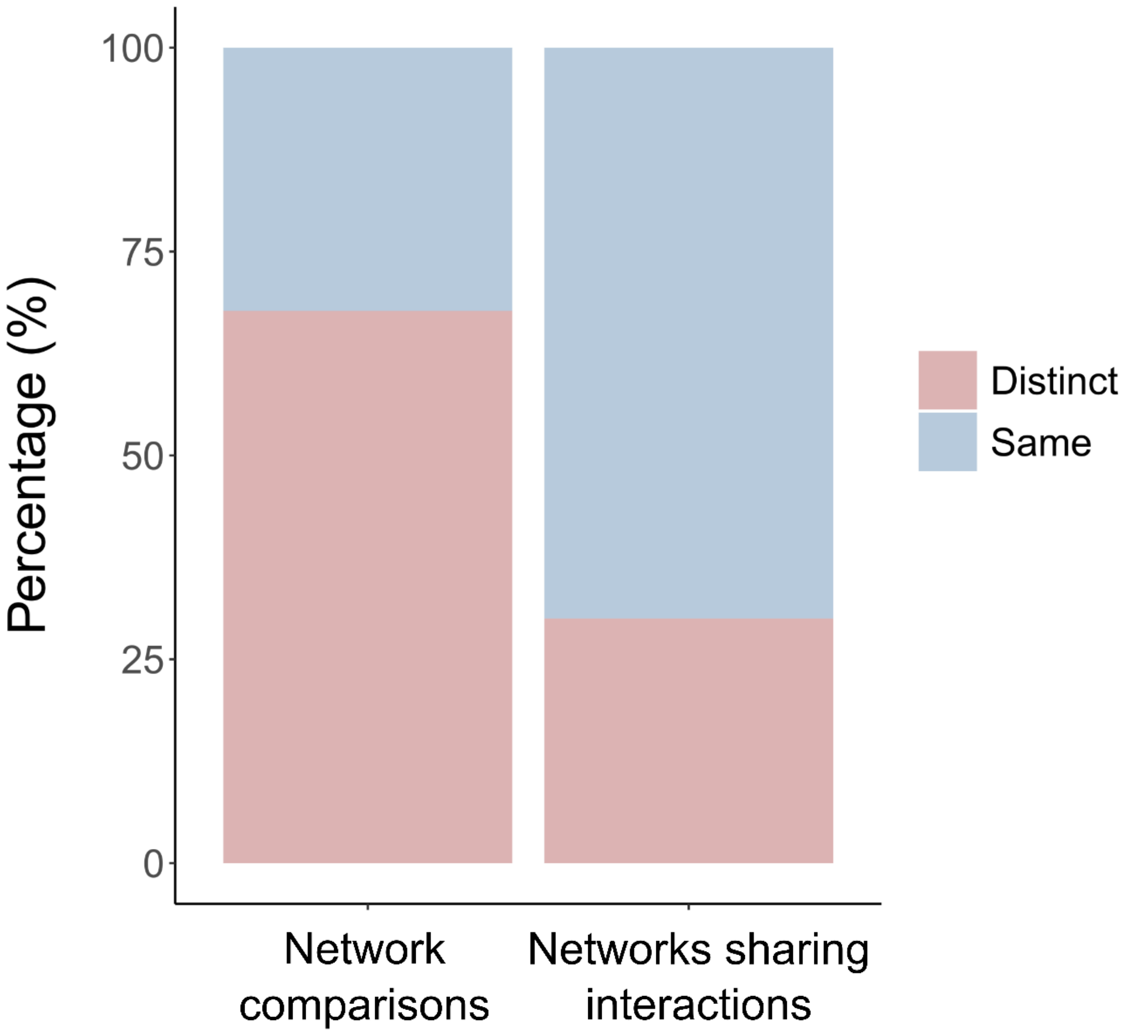

Supplementary Fig. 18. Percentage of long-distance network comparisons and connections (shared interactions) across (‘distinct’) and within (‘same’) biomes. Around 67% of the pairs of networks located >10,000 km of distance from each other (i.e., long-distance network comparisons) involved networks from distinct biomes. On the other hand, 70% of the long-distance connections (i.e., 70% of the pairs of networks that are located > 10,000 km from each other *and* share interactions) involved networks from the same biome.

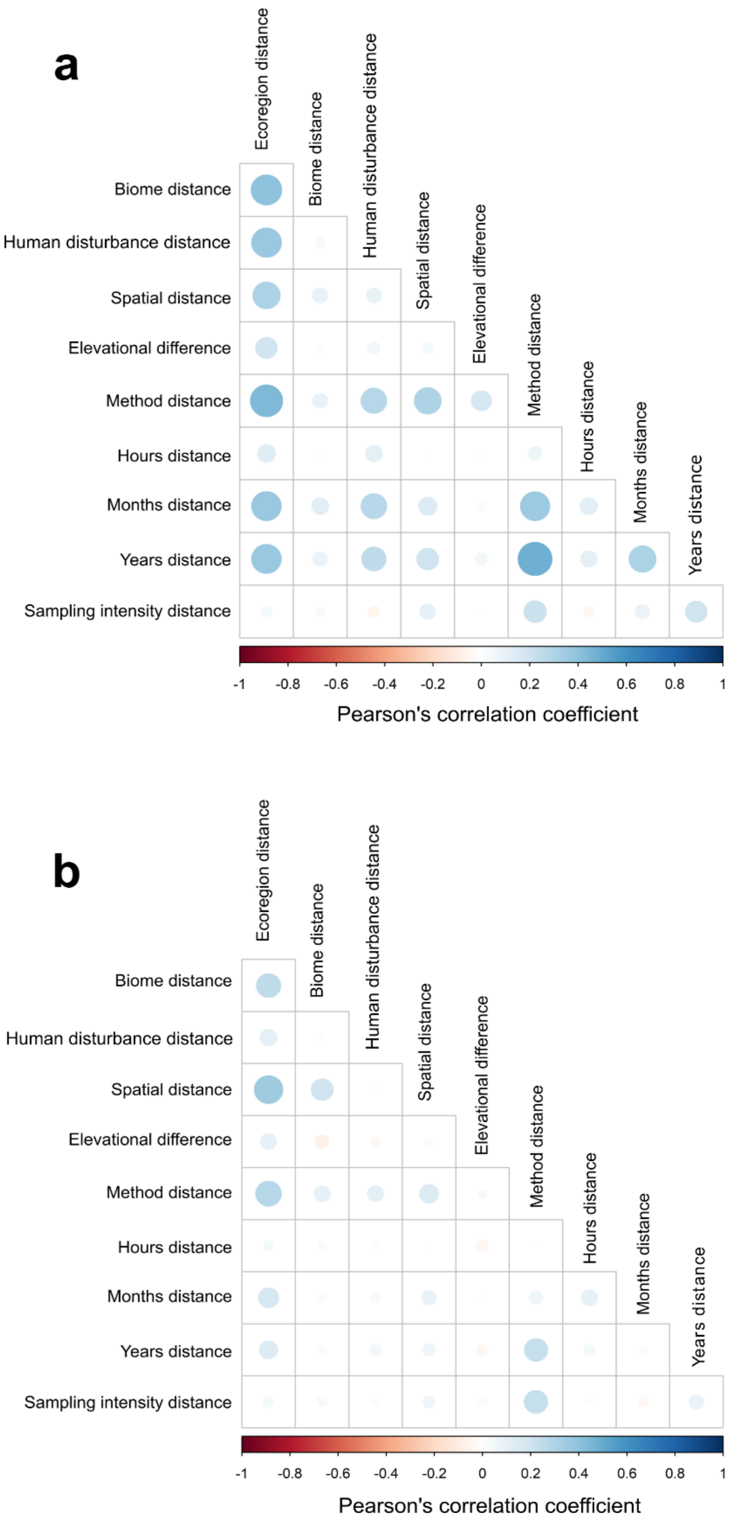

Supplementary Fig. 19. Correlations between the predictor variables used in our models. a Correlations between predictors used in our interaction rewiring analysis. **b** Correlations between predictors used in our model with interaction dissimilarity as the response variable (see Table 1 in the main text). Sizes of the circles and colors are proportional to the correlation coefficient.

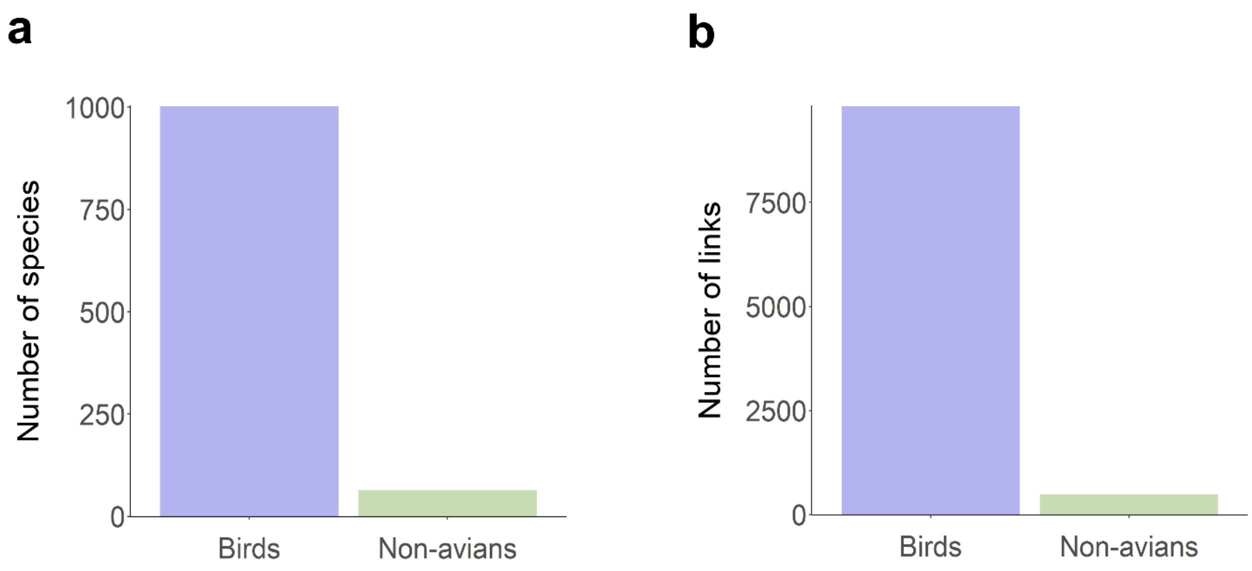

Supplementary Fig. 20. The number of bird and non-avian frugivore species and links (i.e., the frugivore fed on a plant species) in our dataset. The removal of non-avian frugivores from our local networks did not strongly decrease **a** the total number of frugivore species in our dataset, and **b** the number of links in the global network of frugivory.

a

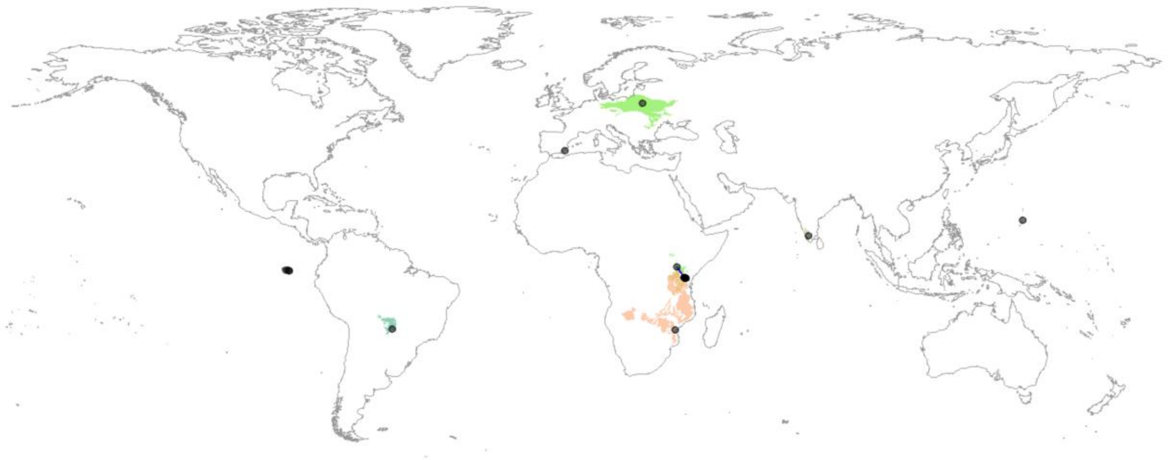

b

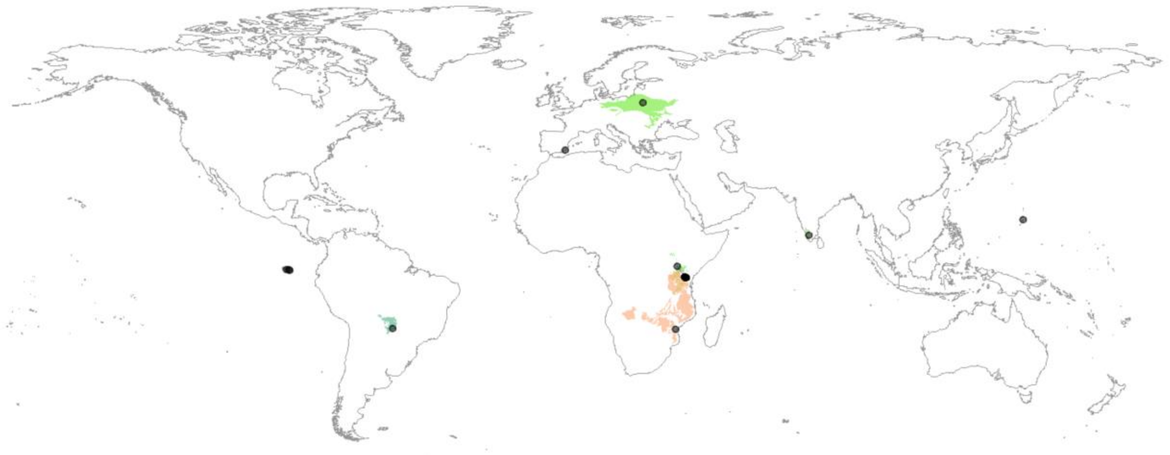

Supplementary Fig. 21. World map with points representing the 28 local networks containing non-avian frugivores in our dataset. Colors of shaded areas represent the nine ecoregions where networks were located. Note the lack of lines representing connections (shared non-avian frugivore species in **a** and shared interactions in **b**, as shown for avian frugivores in Fig. 2 in the main text) between networks located at distinct ecoregions and biomes. Ecoregions and biomes were defined based on the map developed by Dinerstein et al.[19] (available at https://ecoregions.appspot.com/ under a CC-BY 4.0 license).

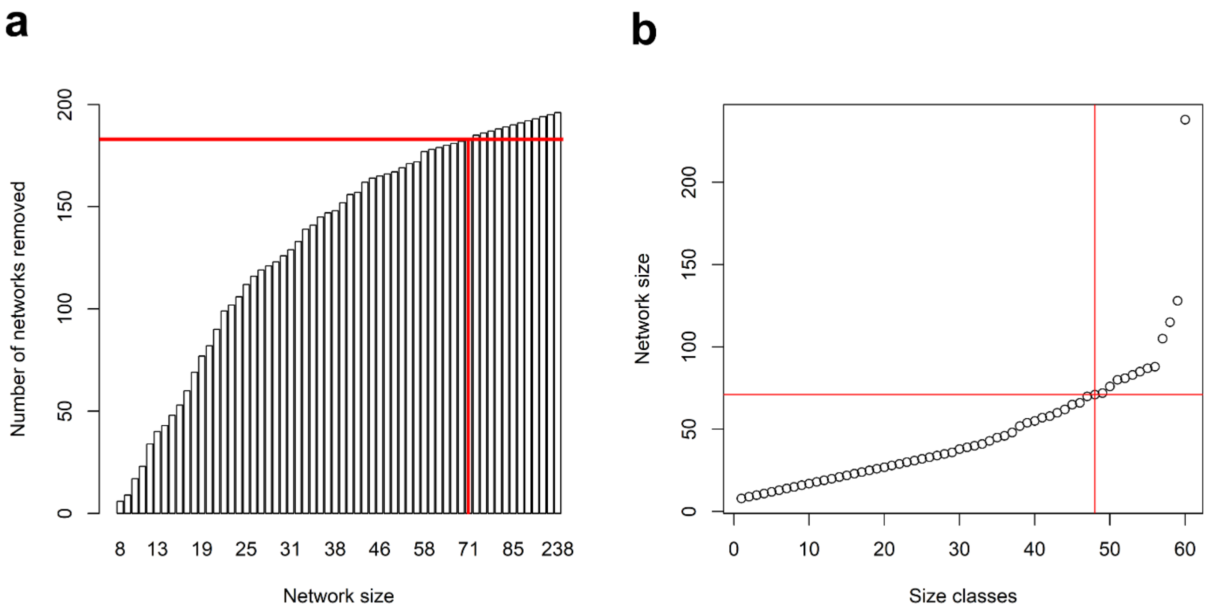

Supplementary Fig. 22. Thresholds used for sequentially removing networks in our sensitivity analysis. a Bars represent the 60 classes of network sizes in our dataset. Numbers below bars indicate the maximum size of the networks within each class. The vertical red line indicates the class of network size up until which analysis could be performed (i.e., after removing all networks up to and including this class; maximum network size = 71 species). The horizontal red line indicates the number of networks removed (*N* networks = 183) in the final round of our sensitivity analysis (i.e., when the maximum network size = 71). **b** Plot showing the number of size classes and network sizes in our dataset. The vertical red line indicates the size class up until which removal could occur and analysis could still be performed (size class rank = 48). The horizontal red line indicates the maximum size (71 species) of networks within this class.

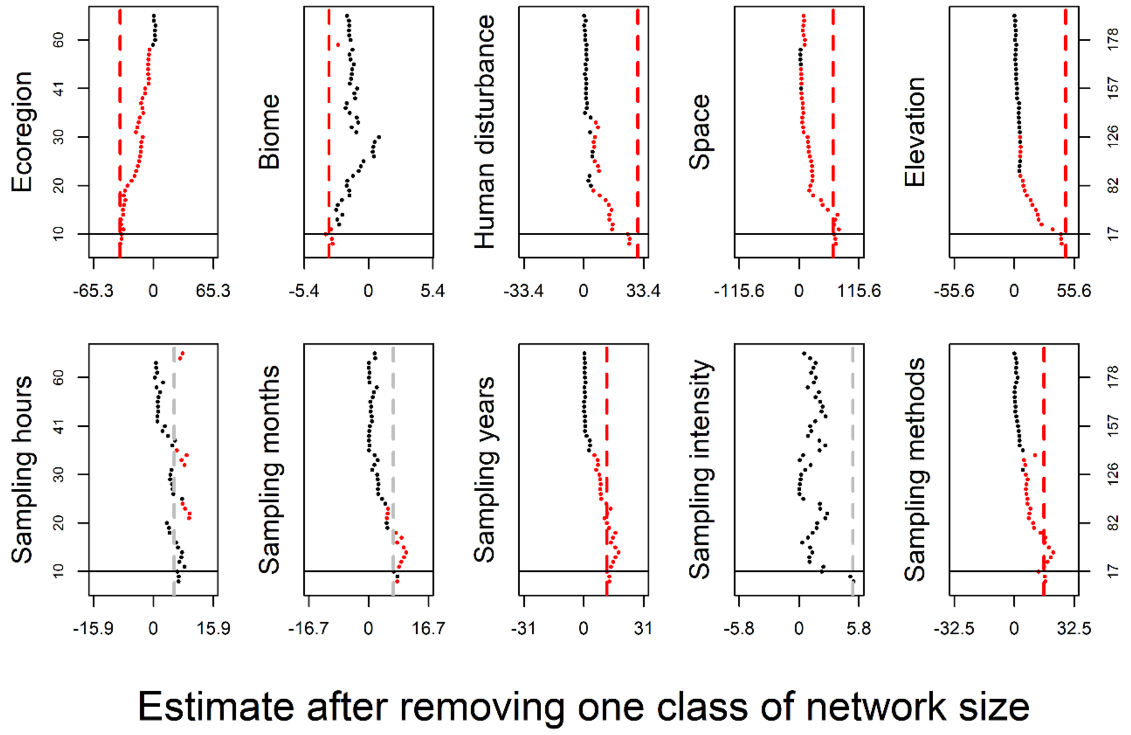

Supplementary Fig. 23. Sensitivity analysis showing the effect of sequentially removing classes of network size on estimates of t (for ecoregion and biome) and F values (for the remaining predictor variables) of Generalized Additive Models with interaction dissimilarity as the response variable. Points represent estimate values after removing all networks below a specified threshold of size [from smallest (bottom) to largest (top) network size; see Supplementary Fig. 22]. Red points indicate a significant effect ($P < 0.05$), while black points indicate a non-significant effect. *P* values were calculated using a combination of Generalized Additive Models and Multiple Regression on distance Matrices (see Methods). The left y-axis represents the threshold network size class below which networks were removed, while the right axis represents the number of networks removed at this threshold. For reference, horizontal black lines indicate the point where networks with up to 10 species (which represented 17 networks) were removed from the dataset. The estimates of the full model (with all networks included) are represented by the vertical lines, with red lines indicating a significant effect and gray lines indicating a non-significant effect. The range of the x-axis was defined as ± 4 times the standard deviation of the estimates (to allow visualization of all estimates). Note that the significant effects in the full model are robust to the removal of small networks (up to 10 species) from the dataset, even though their estimates progressively tend towards zero as larger networks are removed (similarly, *P* values tend to increase and fluctuate around the significance threshold as estimates approach zero). Notably, the effect that seems to be most sensitive to the removal of small networks (i.e., biome distance) explained a low unique proportion of the variation in interaction dissimilarity in our full model, as most of the deviance explained by biome boundaries was shared with ecoregions (Supplementary Fig. 12).

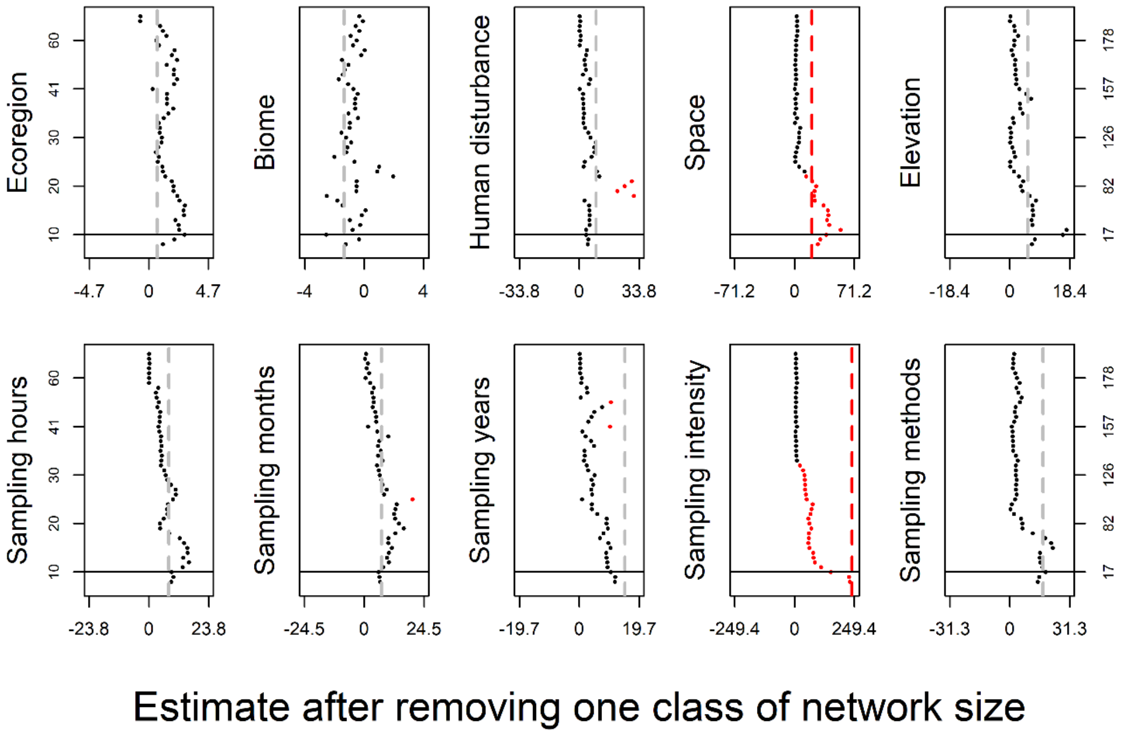

Supplementary Fig. 24. Sensitivity analysis showing the effect of sequentially removing classes of network size on estimates of t (for ecoregion and biome) and F values (for the remaining predictor variables) of Generalized Additive Models with network structural dissimilarity as response variable. Points represent estimate values after removing all networks below a specified threshold of size [from smallest (bottom) to largest (top) network size; see Supplementary Fig. 22]. Red points indicate a significant effect ($P < 0.05$), while black points indicate a non-significant effect. *P* values were calculated using a combination of Generalized Additive Models and Multiple Regression on distance Matrices (see Methods). The left y-axis represents the threshold network size class below which networks were removed, while the right axis represents the number of networks removed at this threshold. For reference, horizontal black lines indicate the point where networks with up to 10 species (which represented 17 networks) were removed from the dataset. The estimates of the full model (with all networks included) are represented by the vertical lines, with red lines indicating a significant effect and gray lines indicating a non-significant effect. The range of the x-axis was defined as ± 4 times the standard deviation of the estimates (to allow visualization of all estimates). Note that the significant effects in the full model (spatial and sampling intensity distances) are very robust to the removal of small networks from the dataset, even though their estimates progressively tend towards zero as larger networks are removed.

Supplementary Table 1. Description of the 196 avian frugivory networks in our dataset. Geographic coordinates were rounded to two decimal places. The metadata of local networks (e.g., original coordinates, sampling methods) are available as Supplementary Data.

Network ID	Latitude	Longitude	Location	Reference
1*	40.33	-74.67	New Jersey, USA	21
2*	18.30	-66.78	Caguana, Puerto Rico	22
3*	18.26	-66.53	Cialitos, Puerto Rico	22
4*	18.17	-66.59	Cordillera, Puerto Rico	22
5*	18.31	-66.56	Fronton, Puerto Rico	22
6*	-28.95	31.75	Mtunzini, South Africa	23
7*	-22.82	-47.11	Mata Santa Genebra, São Paulo, Brazil	24
8*	-22.82	-47.11	Mata Santa Genebra, São Paulo, Brazil	24
9*	18.51	-89.49	Campeche state, Mexico	25
10*	51.77	-1.33	Oxford, United Kingdom	26
11*	-24.32	-48.39	Intervales, São Paulo, Brazil	27
12	-24.13	-47.95	Carlos Botelho, São Paulo, Brazil	28
13	-25.13	-47.96	Ilha do Cardoso, São Paulo, Brazil	29
14	-22.55	-42.28	Poço das Antas, Rio de Janeiro, Brazil	30
15	-23.55	-45.06	Ilha Anchieta, São Paulo, Brazil	31
16	-20.80	-42.86	Viçosa, Minas Gerais, Brazil	32
17	-28.22	-51.17	Estação Aracuri, Rio Grande do Sul, Brazil	33
18	-22.94	-46.75	Itatiba, São Paulo, Brazil	34
19	-22.48	-47.59	Rio Claro, São Paulo, Brazil	35
20	-22.82	-47.43	Santa Barbara do Oeste, São Paulo, Brazil	36
21	-22.67	-47.20	Cosmópolis, São Paulo, Brazil	36
22	-22.57	-47.50	Iracemápolis, São Paulo, Brazil	36
23	-23.55	-46.72	São Paulo, Brazil	37
24	-22.71	-47.61	Piracicaba, São Paulo, Brazil	38
25	-22.77	-43.69	Rio de Janeiro, Brazil	39
26	37.79	-25.18	Azores, Portugal	40
27*	0.30	34.79	Kakamega Forest, Kenya	41
28	-25.49	-49.26	Curitiba, Paraná, Brazil	42
29	-25.44	-49.24	Curitiba, Paraná, Brazil	42
30	-25.44	-49.22	Curitiba, Paraná, Brazil	42
31	-25.42	-49.37	Curitiba, Paraná, Brazil	42
32	-25.41	-49.27	Curitiba, Paraná, Brazil	42
33	-25.36	-49.26	Curitiba, Paraná, Brazil	42
34	-25.38	-49.32	Curitiba, Paraná, Brazil	42
35	-25.17	-48.41	Paraná, Brazil	43
36	28.03	-15.46	Bandama, Gran Canaria, Spain	44
37	28.07	-15.46	El Palomar, Gran Canaria, Spain	44
38	-12.99	-41.34	Chapada Diamantina, Bahia, Brazil	45
39	37.18	-6.32	Hato Ratón, Sevilla, Spain	46
40	-16.40	-67.50	Chulumani, Bolivia	47
41	30.33	130.50	Yakushima Island, Japan	48

Network ID	Latitude	Longitude	Location	Reference
42	-18.95	-48.20	Uberlândia, Minas Gerais, Brazil	[49]
43	21.44	-158.08	Ēkahanui, Hawai'i, USA	[50]
44	21.54	-158.19	Kahanahāiki, Hawai'i, USA	[50]
45	21.38	-157.87	Moanalua, Hawai'i, USA	[50]
46	21.51	-158.14	Mount Ka'ala, Hawai'i, USA	[50]
47	21.54	-158.18	Pahole, Hawai'i, USA	[50]
48	21.34	-157.81	Tantalus, Hawai'i, USA	[50]
49	21.63	-158.04	Waimea Valley, Hawai'i, USA	[50]
50	37.57	-0.91	Sierra de la Fausilla, Murcia, Spain	[51]
51	26.99	92.94	Pakke Tiger Reserve, India	[52]
52	7.77	-76.67	Tulenapa, Antioquia, Colombia	[53]
53	43.28	-5.50	Cantabrian Range, Spain	[54]
54	-29.06	-50.07	Rio Grande do Sul, Brazil	[55]
55	-31.67	-53.25	Rio Grande do Sul, Brazil	[55]
56	15.17	145.77	Saipan, Mariana Islands	[56]
57	14.14	145.21	Rota, Mariana Islands	[56]
58	-0.75	-90.32	Santa Cruz, Galapagos Islands	[57]
59	52.74	23.78	Białowieża Forest, Poland	[58]
60	-4.92	-73.75	Jenaro Herrera, Peru	[59]
61	18.47	-67.11	Finca Montaña, Aguadilla, Puerto Rico	[60]
62	19.59	-96.38	Veracruz, Mexico	[61]
63	-8.97	-36.05	Coimbra Forest, Alagoas, Brazil	[62]
64	-41.29	174.73	Wellington, Aotearoa New Zealand	[63]
65	-41.29	174.75	Wellington, Aotearoa New Zealand	[63]
66	-41.30	174.75	George Denton Park, Aotearoa New Zealand	[63]
67	-41.29	174.80	Charles Plimmer Park, Aotearoa New Zealand	[63]
68	-41.28	174.77	Wellington, Aotearoa New Zealand	[63]
69	-42.35	173.57	Hinau Reserve, Aotearoa New Zealand	[63]
70	-42.33	173.63	Mount Fyffe Reserve, Aotearoa New Zealand	[63]
71	-42.28	173.74	Puhi-Puhi, Aotearoa New Zealand	[63]
72	-42.24	173.78	Blue Duck Reserve, Aotearoa New Zealand	[63]
73	40.22	-8.46	Choupal, Coimbra, Portugal	[64]
74	-41.30	174.75	Wellington, Aotearoa New Zealand	[65]
75	-12.93	-38.40	Salvador, Bahia, Brazil	[66]
76	26.93	92.97	Pakke Tiger Reserve, India	[67]
77	27.02	92.95	Papum Reserve Forest, India	[67]
78	-43.75	169.40	Windbag Valley, Aotearoa New Zealand	[68]
79	37.78	-25.15	Azores, Portugal	[40]
80	37.80	-25.16	Azores, Portugal	[40]
81	37.79	-25.16	Azores, Portugal	[40]
82	40.31	-8.40	Coimbra, Portugal	[69]
83	40.26	-8.48	Coimbra, Portugal	Unpublished[1]
84	-0.66	-90.32	Santa Cruz, Galapagos Islands	[57]
85	-0.91	-89.43	San Cristóbal, Galapagos Islands	[57]
86	-0.89	-89.49	San Cristóbal, Galapagos Islands	[57]

Network ID	Latitude	Longitude	Location	Reference
87	-19.95	34.37	Gorongosa National Park, Mozambique	[70]
88	13.70	80.19	Sriharikota Island, India	[71]
89	-5.05	-37.52	Furna Feia, Rio Grande do Norte, Brazil	[72]
90	-7.22	146.81	Mount Missim, New Guinea	[73]
91	-9.45	147.35	Varirata National Park, New Guinea	[74]
92	-29.12	26.17	Bloemfontein, South Africa	[75]
93	-37.62	144.42	Lerderderg Park, Australia	[76]
94	-37.72	145.57	Mt Healesville and Donna Buang, Australia	[77]
95	-41.33	173.05	Brightwater, Aotearoa New Zealand	[78]
96	-41.32	173.26	Nelson, Aotearoa New Zealand	[78]
97	-41.41	173.04	Faulkners, Wakefield, Aotearoa New Zealand	[78]
98	-22.28	-41.68	Restinga de Jurubatiba, Rio de Janeiro, Brazil	[79]
99	-15.95	-47.97	Brasília, Brazil	[80]
100	-19.77	-40.04	Comboios, Espírito Santo, Brazil	[81]
101	-23.37	-46.60	Cantareira, São Paulo, Brazil	[82]
102	-19.57	-56.20	Pantanal, Brazil	[83]
103	-22.39	-47.54	Rio Claro, São Paulo, Brazil	Unpublished[2]
104	-21.73	-48.02	Araraquara, São Paulo, Brazil	[84]
105	-24.73	-64.67	El Rey National Park, Argentina	[85]
106	-27.25	-65.88	Campo de Los Alisos, Argentina	[85]
107	-27.23	-65.62	La Florida Provincial Park, Argentina	[85]
108	-26.80	-65.30	San Javier y Yerba Huasi, Argentina	[86]
109	-24.76	-64.69	Pozo Verde, El Rey National Park, Argentina	[87]
110	-27.03	-65.77	Quebrada del Portugues, Argentina	[85]
111	-24.10	-64.45	EcoPortal de Piedra, Argentina	[85]
112	-23.69	-64.88	Calilegua National Park, Argentina	[85]
113	-23.69	-64.87	Calilegua National Park, Argentina	[85]
114	-22.28	-64.71	El Nogalar de los Toldos, Argentina	[85]
115	-26.75	-65.33	Parque Sierra de San Javier, Argentina	[87]
116	-26.80	-65.33	Parque Sierra de San Javier, Argentina	Unpublished[3]
117	-15.35	-39.20	Bahia, Brazil	[88]
118	-15.21	-39.14	Bahia, Brazil	[88]
119	-15.13	-39.12	Bahia, Brazil	[88]
120	-15.25	-39.08	Bahia, Brazil	[88]
121	-15.26	-39.09	Bahia, Brazil	[88]
122	10.28	-84.05	Rara Avis Reserve, Costa Rica	[89]
123	-17.85	146.08	Mission Beach, Queensland, Australia	[90]
124	10.35	77.04	Valparai and Anamalai Reserve, India	[91]
125	31.07	103.71	Dujiangyan, Sichuan Province, China	[92]
126	31.05	103.74	Dujiangyan, Sichuan Province, China	[92]
127	31.05	103.73	Dujiangyan, Sichuan Province, China	[92]
128	31.06	103.72	Dujiangyan, Sichuan Province, China	[92]
129	31.05	103.72	Dujiangyan, Sichuan Province, China	[92]
130	31.08	103.70	Dujiangyan, Sichuan Province, China	[92]
131	31.09	103.72	Dujiangyan, Sichuan Province, China	[92]

Network ID	Latitude	Longitude	Location	Reference
132	31.09	103.73	Dujiangyan, Sichuan Province, China	[92]
133	31.08	103.72	Dujiangyan, Sichuan Province, China	[92]
134	31.06	103.73	Dujiangyan, Sichuan Province, China	[92]
135	31.06	103.72	Dujiangyan, Sichuan Province, China	[92]
136	31.05	103.73	Dujiangyan, Sichuan Province, China	[92]
137	31.05	103.73	Dujiangyan, Sichuan Province, China	[92]
138	37.98	-2.90	Serranía de Cazorla, Spain	[93]
139	37.38	-5.71	El Viso del Alcor, Sevilla, Spain	[93]
140	50.30	8.66	Friedberg, Hesse, Germany	[94]
141	51.15	9.00	Kellerwald-Edersee, Germany	[95]
142	-3.23	37.27	Mt Kilimanjaro, Tanzania	[20]
143	-3.25	37.32	Mt Kilimanjaro, Tanzania	[20]
144	-3.27	37.47	Mt Kilimanjaro, Tanzania	[20]
145	-3.17	37.24	Mt Kilimanjaro, Tanzania	[20]
146	-3.21	37.34	Mt Kilimanjaro, Tanzania	[20]
147	-3.26	37.42	Mt Kilimanjaro, Tanzania	[20]
148	-3.26	37.42	Mt Kilimanjaro, Tanzania	[20]
149	-3.23	37.52	Mt Kilimanjaro, Tanzania	[20]
150	-3.14	37.24	Mt Kilimanjaro, Tanzania	[20]
151	-3.13	37.24	Mt Kilimanjaro, Tanzania	[20]
152	-3.14	37.30	Mt Kilimanjaro, Tanzania	[20]
153	-3.14	37.31	Mt Kilimanjaro, Tanzania	[20]
154	-3.17	37.36	Mt Kilimanjaro, Tanzania	[20]
155	-3.15	37.29	Mt Kilimanjaro, Tanzania	[20]
156	-3.18	37.36	Mt Kilimanjaro, Tanzania	[20]
157	-3.19	37.51	Mt Kilimanjaro, Tanzania	[20]
158	-3.20	37.52	Mt Kilimanjaro, Tanzania	[20]
159	-3.19	37.44	Mt Kilimanjaro, Tanzania	[20]
160	-3.10	37.26	Mt Kilimanjaro, Tanzania	[20]
161	-3.17	37.36	Mt Kilimanjaro, Tanzania	[20]
162	-3.16	37.36	Mt Kilimanjaro, Tanzania	[20]
163	-3.19	37.44	Mt Kilimanjaro, Tanzania	[20]
164	-3.18	37.51	Mt Kilimanjaro, Tanzania	[20]
165	-3.18	37.25	Mt Kilimanjaro, Tanzania	[20]
166	-3.30	37.50	Mt Kilimanjaro, Tanzania	[20]
167	-3.33	37.50	Mt Kilimanjaro, Tanzania	[20]
168	-3.30	37.62	Mt Kilimanjaro, Tanzania	[20]
169	-3.19	37.25	Mt Kilimanjaro, Tanzania	[20]
170	-3.27	37.60	Mt Kilimanjaro, Tanzania	[20]
171	-3.32	37.67	Mt Kilimanjaro, Tanzania	[20]
172	-3.37	37.45	Mt Kilimanjaro, Tanzania	[20]
173	-3.38	37.50	Mt Kilimanjaro, Tanzania	[20]
174	-3.33	37.64	Mt Kilimanjaro, Tanzania	[20]
175	-3.32	37.68	Mt Kilimanjaro, Tanzania	[20]
176	-3.31	37.68	Mt Kilimanjaro, Tanzania	[20]

Network ID	Latitude	Longitude	Location	Reference
177	-16.40	-67.50	Chulumani, Bolivia	47
178	4.72	-75.57	Otún Quimbaya, Colombia	96
179	4.70	-75.48	Ucumarí, Colombia	96
180	-3.96	-79.06	Podocarpus National Park, Ecuador	97
181	-4.10	-79.17	Podocarpus National Park, Ecuador	97
182	-13.05	-71.54	San Pedro, Peru	98
183	-13.17	-71.58	Wayqecha, Peru	98
184	9.71	-69.58	Yacambú National Park, Venezuela	99
185	10.39	-67.02	Altos de Pipe, Coastal Cordillera, Venezuela	99
186	10.30	79.85	Point Calimere Wildlife Sanctuary, India	100
187	20.60	-156.33	Kanaio Natural Area Reserve, Hawai'i	101
188	-3.37	38.33	Taita Hills, Kenya	102
189	40.13	-88.17	Champaign County, Illinois, USA	103
190	-17.53	-149.83	Moorea, French Polynesia	104
191	10.47	-83.51	Tortuguero, Costa Rica	105
192	24.80	121.25	Fushan Experimental Forest, Taiwan	106
193	22.46	91.77	Chittagong, Bangladesh	107
194	10.42	-84.01	La Selva Biological Station, Costa Rica	108
195	10.42	-84.02	La Selva Biological Station, Costa Rica	109
196	39.14	2.94	Cabrera Island, Spain	110

*Obtained through the Web of Life database[111].
Unpublished[1]: Data provided by Ruben Heleno.
Unpublished[2]: Data provided by Marco Aurélio Pizo.
Unpublished[3]: Data provided by Pedro G. Blendinger.

Supplementary Table 2. Quantitative metrics of network sampling. Sampling intensity and completeness aim to account for how complete network sampling was in terms of species interactions, while sampling hours and months account for the time-span of the study.

Sampling metric	Rationale
Sampling intensity	Sampling intensity was calculated as the square-root of the number of interaction events divided by the square-root of the product of the number of plant and animal species in the local network[112]. Sampling intensity was included in our models because it presented a strong and positive relationship with the ratio between the number of interactions sampled in the local network and the number of known possible interactions (among that same set of species) in the region (for the subset of networks within the Aotearoa New Zealand meta-network) (Supplementary Fig. 7).
Sampling completeness	Sampling completeness was calculated as the observed richness of links divided by the estimated richness of links in the local network[113]. We used the Chao 1 richness estimator[114] to obtain the estimated number of links in our networks. Sampling completeness was not included in our models because it did not present a significant relationship with the ratio between the number of interactions in the local network and the number of known possible interactions (among that same set of species) in the region (Supplementary Fig. 7). Thus, we considered that this metric did not provide a good representation of how complete network sampling was in terms of species interactions.
Sampling hours	Number of sampling hours was included in our statistical models because it presented strong and positive relationships with bird richness, plant richness and number of links in the local networks (Supplementary Fig. 8).
Sampling months	Number of sampling months was included in our statistical models because it presented a strong and positive relationship with the ratio between the number of interactions in the local network and the number of known possible interactions (among that same set of species) in the region (Supplementary Fig. 7), as well as with plant richness and number of links in the local networks (for the entire dataset) (Supplementary Fig. 8).

Supplementary Table 3. Description of variables used to generate the method's dissimilarity matrix.

Variable	Description
Sampling design	Whether the sampling design was 'transect', 'plot', 'mist-net', 'focal observation', 'camera-trap', or any combination of these.
Sampling focus	Whether the focal organisms were birds, plants, or both. As such, this variable determines if authors used a zoocentric or a phytocentric sampling method (or a combination of the two).
Sampling coverage	Whether there were focal species ('partial coverage') or not ('total coverage').
Interaction frequency type	Whether interaction frequency was estimated by counting the number of bird visits, number of fruits consumed by the bird, number of seeds in bird droppings, or number of bird droppings with seeds.

Supplementary Table 4. Multiple predictors of species turnover (β_S) on plant-frugivore networks. Here, we used the binary version of ecoregion and biome distance matrices. *P* values were calculated using a two-tailed statistical test that combines Generalized Additive Models (GAM) and Multiple Regression on distance Matrices (MRM). In this approach, the non-independence of distances from each local network is accounted for in the hypothesis testing by performing 1,000 permutations of the response matrix (see Methods). EDF represents the estimated degrees of freedom for each smooth term in the model. *N* pairs of networks = 19,110.

**Parametric coefficients**	**Estimate**	**t**	**P**
Intercept	0.976	1734.300	**0.001**
Ecoregion (same)	-0.122	-38.093	**0.001**
Biome (same)	-0.008	-8.799	**0.001**
**Smooth Terms**	**EDF**	**F**	**P**
s (human disturbance distance)	8.312	28.504	**0.001**
s (spatial distance)	8.866	725.571	**0.001**
s (elevational difference)	5.589	99.954	**0.001**
s (hours distance)	6.917	4.004	0.619
s (months distance)	6.755	6.525	0.089
s (years distance)	6.402	7.422	0.068
s (sampling intensity distance)	1.007	26.580	**0.005**
s (methods distance)	8.039	10.911	**0.015**

Bold values indicate statistically significant results ($P < 0.05$).

Supplementary Table 5. Multiple predictors of species turnover (β_S) on plant-frugivore networks. Here, we used the quantitative version (environmental dissimilarity) of ecoregion and biome distance matrices. *P* values were calculated using a two-tailed statistical test that combines Generalized Additive Models (GAM) and Multiple Regression on distance Matrices (MRM). In this approach, the non-independence of distances from each local network is accounted for in the hypothesis testing by performing 1,000 permutations of the response matrix (see Methods). EDF represents the estimated degrees of freedom for each smooth term in the model. *N* pairs of networks = 19,110.

Smooth Terms	EDF	F	P
s (ecoregion distance)	8.570	137.969	**0.001**
s (biome distance)	8.202	37.937	**0.001**
s (human disturbance distance)	8.339	29.465	**0.001**
s (spatial distance)	8.890	698.382	**0.001**
s (elevational difference)	5.517	98.173	**0.001**
s (hours distance)	7.330	4.876	0.448
s (months distance)	5.371	5.811	0.109
s (years distance)	6.152	7.741	0.063
s (sampling intensity distance)	4.365	6.108	0.315
s (methods distance)	7.996	11.474	**0.017**

Bold values indicate statistically significant results ($P < 0.05$).

Supplementary Table 6. Multiple predictors of plant-frugivore interaction dissimilarity (β_{WN}). Here, we used the quantitative version (environmental dissimilarity) of ecoregion and biome distance matrices. *P* values were calculated using a two-tailed statistical test that combines Generalized Additive Models (GAM) and Multiple Regression on distance Matrices (MRM). In this approach, the non-independence of distances from each local network is accounted for in the hypothesis testing by performing 1,000 permutations of the response matrix (see Methods). EDF represents the estimated degrees of freedom for each smooth term in the model. ($P < 0.05$). *N* pairs of networks = 19,110.

Smooth Terms	EDF	F	P
s (ecoregion distance)	8.595	110.122	**0.001**
s (biome distance)	7.827	10.492	**0.022**
s (human disturbance distance)	8.570	32.573	**0.001**
s (spatial distance)	8.855	81.843	**0.001**
s (elevational difference)	6.024	48.426	**0.001**
s (hours distance)	1.353	10.637	**0.043**
s (months distance)	5.800	7.876	**0.045**
s (years distance)	7.135	13.007	**0.020**
s (sampling intensity distance)	1.010	5.437	0.267
s (methods distance)	7.878	17.094	**0.003**

Bold values indicate statistically significant results ($P < 0.05$).

Supplementary Table 7. Multiple predictors of plant-frugivore network structural dissimilarity. Here, we used the binary version of ecoregion and biome distance matrices. *P* values were calculated using a two-tailed statistical test that combines Generalized Additive Models (GAM) and Multiple Regression on distance Matrices (MRM). In this approach, the non-independence of distances from each local network is accounted for in the hypothesis testing by performing 1,000 permutations of the response matrix (see Methods). EDF represents the estimated degrees of freedom for each smooth term in the model. *N* pairs of networks = 19,110.

**Parametric coefficients**	**Estimate**	**t**	**P**
Intercept	2.689	222.572	**0.002**
Ecoregion (same)	0.043	0.632	0.788
Biome (same)	-0.028	-1.345	0.770
**Smooth Terms**	**EDF**	**F**	**P**
s (human disturbance distance)	5.923	9.346	0.429
s (spatial distance)	8.474	20.408	**0.021**
s (elevational difference)	8.220	5.510	0.749
s (hours distance)	8.006	7.944	0.969
s (months distance)	5.961	7.078	0.693
s (years distance)	6.868	14.999	0.461
s (sampling intensity distance)	8.762	238.987	**0.002**
s (methods distance)	8.586	17.372	0.231

Bold values indicate statistically significant results ($P < 0.05$).

Supplementary Table 8. Multiple predictors of plant-frugivore network structural dissimilarity. Here, we used the quantitative version (environmental dissimilarity) of ecoregion and biome distance matrices. *P* values were calculated using a two-tailed statistical test that combines Generalized Additive Models (GAM) and Multiple Regression on distance Matrices (MRM). In this approach, the non-independence of distances from each local network is accounted for in the hypothesis testing by performing 1,000 permutations of the response matrix (see Methods). EDF represents the estimated degrees of freedom for each smooth term in the model. *N* pairs of networks = 19,110.

**Smooth Terms**	**EDF**	**F**	**P**
s (ecoregion distance)	4.272	15.275	0.193
s (biome distance)	7.697	12.115	0.568
s (human disturbance distance)	5.993	9.264	0.438
s (spatial distance)	8.465	18.465	**0.018**
s (elevational difference)	8.290	5.679	0.713
s (hours distance)	7.857	8.913	0.955
s (months distance)	6.173	8.239	0.606
s (years distance)	6.751	12.872	0.545
s (sampling intensity distance)	8.760	239.475	**0.002**
s (methods distance)	8.501	15.584	0.257

Bold values indicate statistically significant results ($P < 0.05$).

Supplementary Table 9. **Multiple predictors of species turnover (β_S) on plant-frugivore networks.** Here, we used a buffer zone of 500 km and the alternative scenario 1 (see Alternative scenarios section) during the data cleaning process. The binary versions of ecoregion and biome distance matrices were used for estimating the effects of ecoregion and biome borders on the response variable. *P* values were calculated using a two-tailed statistical test that combines Generalized Additive Models (GAM) and Multiple Regression on distance Matrices (MRM). In this approach, the non-independence of distances from each local network is accounted for in the hypothesis testing by performing 1,000 permutations of the response matrix (see Methods). EDF represents the estimated degrees of freedom for each smooth term in the model. *N* pairs of networks = 19,110.

**Parametric coefficients**	**Estimate**	**t**	**P**
Intercept	0.976	1735.328	**0.001**
Ecoregion (same)	-0.122	-38.147	**0.001**
Biome (same)	-0.008	-8.809	**0.001**
**Smooth Terms**	**EDF**	**F**	**P**
s (human disturbance distance)	8.312	28.538	**0.001**
s (spatial distance)	8.867	725.453	**0.001**
s (elevational difference)	5.600	99.711	**0.001**
s (hours distance)	6.928	4.042	0.580
s (months distance)	6.761	6.566	0.083
s (years distance)	6.412	7.472	0.059
s (sampling intensity distance)	1.001	26.885	**0.005**
s (methods distance)	8.032	10.833	**0.023**

Bold values indicate statistically significant results ($P < 0.05$).

Supplementary Table 10. **Multiple predictors of species turnover (β_S) on plant-frugivore networks.** Here, we used a buffer zone of 500 km and the alternative scenario 2 (see Alternative scenarios section) during the data cleaning process. The binary versions of ecoregion and biome distance matrices were used for estimating the effects of ecoregion and biome borders on the response variable. *P* values were calculated using a two-tailed statistical test that combines Generalized Additive Models (GAM) and Multiple Regression on distance Matrices (MRM). In this approach, the non-independence of distances from each local network is accounted for in the hypothesis testing by performing 1,000 permutations of the response matrix (see Methods). EDF represents the estimated degrees of freedom for each smooth term in the model. *N* pairs of networks = 19,110.

**Parametric coefficients**	**Estimate**	**t**	**P**
Intercept	0.976	1752.859	**0.001**
Ecoregion (same)	-0.123	-38.615	**0.001**
Biome (same)	-0.008	-8.084	**0.001**
**Smooth Terms**	**EDF**	**F**	**P**
s (human disturbance distance)	8.437	28.851	**0.001**
s (spatial distance)	8.865	719.288	**0.001**
s (elevational difference)	5.600	99.486	**0.001**
s (hours distance)	7.126	4.330	0.559
s (months distance)	4.001	6.532	0.091
s (years distance)	6.548	8.206	0.069
s (sampling intensity distance)	3.464	8.113	0.166
s (methods distance)	8.114	11.641	**0.013**

Bold values indicate statistically significant results ($P < 0.05$).

Supplementary Table 11. **Multiple predictors of species turnover (β_S) on plant-frugivore networks.** Here, we used a buffer zone of 100 km and the alternative scenario 1 (see Alternative scenarios section) during the data cleaning process. The binary versions of ecoregion and biome distance matrices were used for estimating the effects of ecoregion and biome borders on the response variable. *P* values were calculated using a two-tailed statistical test that combines Generalized Additive Models (GAM) and Multiple Regression on distance Matrices (MRM). In this approach, the non-independence of distances from each local network is accounted for in the hypothesis testing by performing 1,000 permutations of the response matrix (see Methods). EDF represents the estimated degrees of freedom for each smooth term in the model. *N* pairs of networks = 19,110.

**Parametric coefficients**	**Estimate**	**t**	**P**
Intercept	0.976	1736.530	**0.001**
Ecoregion (same)	-0.122	-38.181	**0.001**
Biome (same)	-0.009	-8.781	**0.001**
**Smooth Terms**	**EDF**	**F**	**P**
s (human disturbance distance)	8.317	28.664	**0.002**
s (spatial distance)	8.866	725.286	**0.001**
s (elevational difference)	5.606	99.783	**0.001**
s (hours distance)	6.888	3.931	0.606
s (months distance)	6.827	6.601	0.091
s (years distance)	6.406	7.500	0.073
s (sampling intensity distance)	1.002	26.760	**0.008**
s (methods distance)	8.029	10.895	**0.016**

Bold values indicate statistically significant results ($P < 0.05$).

Supplementary Table 12. **Multiple predictors of species turnover (β_S) on plant-frugivore networks.** Here, we used a buffer zone of 100 km and the alternative scenario 2 (see Alternative scenarios section) during the data cleaning process. The binary versions of ecoregion and biome distance matrices were used for estimating the effects of ecoregion and biome borders on the response variable. *P* values were calculated using a two-tailed statistical test that combines Generalized Additive Models (GAM) and Multiple Regression on distance Matrices (MRM). In this approach, the non-independence of distances from each local network is accounted for in the hypothesis testing by performing 1,000 permutations of the response matrix (see Methods). EDF represents the estimated degrees of freedom for each smooth term in the model. *N* pairs of networks = 19,110.

**Parametric coefficients**	**Estimate**	**t**	**P**
Intercept	0.976	1755.726	**0.001**
Ecoregion (same)	-0.122	-38.561	**0.001**
Biome (same)	-0.008	-8.354	**0.001**
**Smooth Terms**	**EDF**	**F**	**P**
s (human disturbance distance)	8.341	29.073	**0.002**
s (spatial distance)	8.863	716.735	**0.001**
s (elevational difference)	5.578	100.041	**0.001**
s (hours distance)	6.987	3.990	0.592
s (months distance)	6.819	6.693	0.107
s (years distance)	6.484	7.966	0.063
s (sampling intensity distance)	1.000	24.580	**0.005**
s (methods distance)	8.013	11.066	**0.018**

Bold values indicate statistically significant results ($P < 0.05$).

Supplementary Table 13. Multiple predictors of species turnover (β_S) on plant-frugivore networks. Here, we used a buffer zone of 100 km and the alternative scenario 3 (see Alternative scenarios section) during the data cleaning process. The binary versions of ecoregion and biome distance matrices were used for estimating the effects of ecoregion and biome borders on the response variable. *P* values were calculated using a two-tailed statistical test that combines Generalized Additive Models (GAM) and Multiple Regression on distance Matrices (MRM). In this approach, the non-independence of distances from each local network is accounted for in the hypothesis testing by performing 1,000 permutations of the response matrix (see Methods). EDF represents the estimated degrees of freedom for each smooth term in the model. *N* pairs of networks = 19,110.

**Parametric coefficients**	**Estimate**	**t**	**P**
Intercept	0.976	1735.345	**0.001**
Ecoregion (same)	-0.122	-38.157	**0.001**
Biome (same)	-0.009	-8.775	**0.001**
**Smooth Terms**	**EDF**	**F**	**P**
s (human disturbance distance)	8.317	28.665	**0.002**
s (spatial distance)	8.866	723.914	**0.001**
s (elevational difference)	5.587	99.935	**0.001**
s (hours distance)	6.918	4.014	0.605
s (months distance)	6.783	6.589	0.100
s (years distance)	6.406	7.492	0.078
s (sampling intensity distance)	1.000	26.866	**0.004**
s (methods distance)	8.033	10.910	**0.011**

Bold values indicate statistically significant results ($P < 0.05$).

Supplementary Table 14. **Multiple predictors of species turnover (β_S) on plant-frugivore networks.** Here, we used a buffer zone of 1000 km and the alternative scenario 1 (see Alternative scenarios section) during the data cleaning process. The binary versions of ecoregion and biome distance matrices were used for estimating the effects of ecoregion and biome borders on the response variable. *P* values were calculated using a two-tailed statistical test that combines Generalized Additive Models (GAM) and Multiple Regression on distance Matrices (MRM). In this approach, the non-independence of distances from each local network is accounted for in the hypothesis testing by performing 1,000 permutations of the response matrix (see Methods). EDF represents the estimated degrees of freedom for each smooth term in the model. *N* pairs of networks = 19,110.

**Parametric coefficients**	**Estimate**	**t**	**P**
Intercept	0.976	1734.871	**0.001**
Ecoregion (same)	-0.122	-38.147	**0.001**
Biome (same)	-0.009	-8.789	**0.001**
**Smooth Terms**	**EDF**	**F**	**P**
s (human disturbance distance)	8.309	28.531	**0.001**
s (spatial distance)	8.866	725.141	**0.001**
s (elevational difference)	5.605	99.321	**0.001**
s (hours distance)	6.911	4.049	0.602
s (months distance)	6.761	6.579	0.099
s (years distance)	6.414	7.440	0.067
s (sampling intensity distance)	1.002	26.590	**0.005**
s (methods distance)	8.030	10.869	**0.019**

Bold values indicate statistically significant results ($P < 0.05$).

Supplementary Table 15. **Multiple predictors of species turnover (β_S) on plant-frugivore networks.** Here, we used a buffer zone of 1000 km and the alternative scenario 2 (see Alternative scenarios section) during the data cleaning process. The binary versions of ecoregion and biome distance matrices were used for estimating the effects of ecoregion and biome borders on the response variable. *P* values were calculated using a two-tailed statistical test that combines Generalized Additive Models (GAM) and Multiple Regression on distance Matrices (MRM). In this approach, the non-independence of distances from each local network is accounted for in the hypothesis testing by performing 1,000 permutations of the response matrix (see Methods). EDF represents the estimated degrees of freedom for each smooth term in the model. *N* pairs of networks = 19,110.

**Parametric coefficients**	**Estimate**	**t**	**P**
Intercept	0.976	1755.726	**0.001**
Ecoregion (same)	-0.122	-38.561	**0.001**
Biome (same)	-0.008	-8.354	**0.001**
**Smooth Terms**	**EDF**	**F**	**P**
s (human disturbance distance)	8.341	29.073	**0.001**
s (spatial distance)	8.863	716.735	**0.001**
s (elevational difference)	5.578	100.041	**0.001**
s (hours distance)	6.987	3.990	0.608
s (months distance)	6.819	6.693	0.087
s (years distance)	6.484	7.966	0.061
s (sampling intensity distance)	1.000	24.580	**0.008**
s (methods distance)	8.013	11.066	**0.016**

Bold values indicate statistically significant results ($P < 0.05$).

Supplementary Table 16. **Multiple predictors of species turnover (β_S) on plant-frugivore networks.** Here, we used a buffer zone of 1000 km and the alternative scenario 3 (see Alternative scenarios section) during the data cleaning process. The binary versions of ecoregion and biome distance matrices were used for estimating the effects of ecoregion and biome borders on the response variable. *P* values were calculated using a two-tailed statistical test that combines Generalized Additive Models (GAM) and Multiple Regression on distance Matrices (MRM). In this approach, the non-independence of distances from each local network is accounted for in the hypothesis testing by performing 1,000 permutations of the response matrix (see Methods). EDF represents the estimated degrees of freedom for each smooth term in the model. *N* pairs of networks = 19,110.

**Parametric coefficients**	**Estimate**	**t**	**P**
Intercept	0.976	1733.860	**0.001**
Ecoregion (same)	-0.122	-38.095	**0.001**
Biome (same)	-0.009	-8.778	**0.001**
**Smooth Terms**	**EDF**	**F**	**P**
s (human disturbance distance)	8.308	28.497	**0.001**
s (spatial distance)	8.866	725.333	**0.001**
s (elevational difference)	5.594	99.561	**0.001**
s (hours distance)	6.899	4.011	0.608
s (months distance)	6.744	6.537	0.109
s (years distance)	6.404	7.389	0.063
s (sampling intensity distance)	1.004	26.506	**0.006**
s (methods distance)	8.037	10.951	**0.021**

Bold values indicate statistically significant results ($P < 0.05$).

Supplementary Table 17. **Multiple predictors of plant-frugivore interaction dissimilarity (β_{WN}).** Here, we used a buffer zone of 500 km and the alternative scenario 1 (see Alternative scenarios section) during the data cleaning process. The binary versions of ecoregion and biome distance matrices were used for estimating the effects of ecoregion and biome borders on the response variable. *P* values were calculated using a two-tailed statistical test that combines Generalized Additive Models (GAM) and Multiple Regression on distance Matrices (MRM). In this approach, the non-independence of distances from each local network is accounted for in the hypothesis testing by performing 1,000 permutations of the response matrix (see Methods). EDF represents the estimated degrees of freedom for each smooth term in the model. *N* pairs of networks = 19,110.

**Parametric coefficients**	**Estimate**	**t**	**P**
Intercept	0.997	2966.347	**0.001**
Ecoregion (same)	-0.070	-36.417	**0.001**
Biome (same)	-0.002	-3.317	**0.039**
**Smooth Terms**	**EDF**	**F**	**P**
s (human disturbance distance)	8.536	30.035	**0.001**
s (spatial distance)	8.785	65.220	**0.001**
s (elevational difference)	6.185	47.606	**0.001**
s (hours distance)	1.545	5.545	0.294
s (months distance)	5.502	6.966	0.074
s (years distance)	7.216	11.880	**0.013**
s (sampling intensity distance)	1.062	4.686	0.331
s (methods distance)	7.848	15.987	**0.004**

Bold values indicate statistically significant results (*P* < 0.05).

Supplementary Table 18. **Multiple predictors of plant-frugivore interaction dissimilarity (β_{WN}).** Here, we used a buffer zone of 500 km and the alternative scenario 2 (see Alternative scenarios section) during the data cleaning process. The binary versions of ecoregion and biome distance matrices were used for estimating the effects of ecoregion and biome borders on the response variable. *P* values were calculated using a two-tailed statistical test that combines Generalized Additive Models (GAM) and Multiple Regression on distance Matrices (MRM). In this approach, the non-independence of distances from each local network is accounted for in the hypothesis testing by performing 1,000 permutations of the response matrix (see Methods). EDF represents the estimated degrees of freedom for each smooth term in the model. *N* pairs of networks = 19,110.

**Parametric coefficients**	**Estimate**	**t**	**P**
Intercept	0.997	3002.392	**0.001**
Ecoregion (same)	-0.069	-36.473	**0.001**
Biome (same)	-0.002	-3.313	**0.034**
**Smooth Terms**	**EDF**	**F**	**P**
s (human disturbance distance)	8.551	30.504	**0.001**
s (spatial distance)	8.783	64.233	**0.001**
s (elevational difference)	6.107	47.553	**0.001**
s (hours distance)	1.590	5.325	0.307
s (months distance)	5.475	7.030	0.092
s (years distance)	7.216	11.941	**0.022**
s (sampling intensity distance)	1.003	5.041	0.319
s (methods distance)	7.867	16.082	**0.003**

Bold values indicate statistically significant results ($P < 0.05$).

Supplementary Table 19. **Multiple predictors of plant-frugivore interaction dissimilarity (β_{WN}).** Here, we used a buffer zone of 100 km and the alternative scenario 1 (see Alternative scenarios section) during the data cleaning process. The binary versions of ecoregion and biome distance matrices were used for estimating the effects of ecoregion and biome borders on the response variable. *P* values were calculated using a two-tailed statistical test that combines Generalized Additive Models (GAM) and Multiple Regression on distance Matrices (MRM). In this approach, the non-independence of distances from each local network is accounted for in the hypothesis testing by performing 1,000 permutations of the response matrix (see Methods). EDF represents the estimated degrees of freedom for each smooth term in the model. *N* pairs of networks = 19,110.

**Parametric coefficients**	**Estimate**	**t**	**P**
Intercept	0.997	2966.503	**0.001**
Ecoregion (same)	-0.070	-36.418	**0.001**
Biome (same)	-0.002	-3.321	**0.047**
**Smooth Terms**	**EDF**	**F**	**P**
s (human disturbance distance)	8.536	30.011	**0.001**
s (spatial distance)	8.785	65.161	**0.001**
s (elevational difference)	6.190	47.625	**0.001**
s (hours distance)	1.546	5.546	0.272
s (months distance)	5.504	6.965	0.074
s (years distance)	7.215	11.883	**0.021**
s (sampling intensity distance)	1.056	4.744	0.330
s (methods distance)	7.851	16.023	**0.005**

Bold values indicate statistically significant results (*P* < 0.05).

Supplementary Table 20. **Multiple predictors of plant-frugivore interaction dissimilarity (β_{WN}).** Here, we used a buffer zone of 100 km and the alternative scenario 2 (see Alternative scenarios section) during the data cleaning process. The binary versions of ecoregion and biome distance matrices were used for estimating the effects of ecoregion and biome borders on the response variable. *P* values were calculated using a two-tailed statistical test that combines Generalized Additive Models (GAM) and Multiple Regression on distance Matrices (MRM). In this approach, the non-independence of distances from each local network is accounted for in the hypothesis testing by performing 1,000 permutations of the response matrix (see Methods). EDF represents the estimated degrees of freedom for each smooth term in the model. *N* pairs of networks = 19,110.

**Parametric coefficients**	**Estimate**	**t**	**P**
Intercept	0.997	3002.382	**0.001**
Ecoregion (same)	-0.069	-36.474	**0.001**
Biome (same)	-0.002	-3.312	**0.049**
**Smooth Terms**	**EDF**	**F**	**P**
s (human disturbance distance)	8.551	30.506	**0.002**
s (spatial distance)	8.782	64.153	**0.001**
s (elevational difference)	6.109	47.538	**0.001**
s (hours distance)	1.579	5.376	0.298
s (months distance)	5.483	7.037	0.075
s (years distance)	7.217	11.954	**0.019**
s (sampling intensity distance)	1.003	5.036	0.311
s (methods distance)	7.867	16.089	**0.004**

Bold values indicate statistically significant results (*P* < 0.05).

Supplementary Table 21. Multiple predictors of plant-frugivore interaction dissimilarity (β_{WN}). Here, we used a buffer zone of 100 km and the alternative scenario 3 (see Alternative scenarios section) during the data cleaning process. The binary versions of ecoregion and biome distance matrices were used for estimating the effects of ecoregion and biome borders on the response variable. *P* values were calculated using a two-tailed statistical test that combines Generalized Additive Models (GAM) and Multiple Regression on distance Matrices (MRM). In this approach, the non-independence of distances from each local network is accounted for in the hypothesis testing by performing 1,000 permutations of the response matrix (see Methods). EDF represents the estimated degrees of freedom for each smooth term in the model. *N* pairs of networks = 19,110.

**Parametric coefficients**	**Estimate**	**t**	**P**
Intercept	0.997	2964.236	**0.001**
Ecoregion (same)	-0.070	-36.405	**0.001**
Biome (same)	-0.002	-3.324	**0.046**
**Smooth Terms**	**EDF**	**F**	**P**
s (human disturbance distance)	8.534	29.980	**0.001**
s (spatial distance)	8.785	65.228	**0.001**
s (elevational difference)	6.171	47.691	**0.001**
s (hours distance)	1.559	5.453	0.301
s (months distance)	5.490	6.908	0.076
s (years distance)	7.210	11.881	**0.020**
s (sampling intensity distance)	1.022	5.148	0.281
s (methods distance)	7.850	16.024	**0.004**

Bold values indicate statistically significant results ($P < 0.05$).

Supplementary Table 22. Multiple predictors of plant-frugivore interaction dissimilarity (β_{WN}). Here, we used a buffer zone of 1000 km and the alternative scenario 1 (see Alternative scenarios section) during the data cleaning process. The binary versions of ecoregion and biome distance matrices were used for estimating the effects of ecoregion and biome borders on the response variable. *P* values were calculated using a two-tailed statistical test that combines Generalized Additive Models (GAM) and Multiple Regression on distance Matrices (MRM). In this approach, the non-independence of distances from each local network is accounted for in the hypothesis testing by performing 1,000 permutations of the response matrix (see Methods). EDF represents the estimated degrees of freedom for each smooth term in the model. *N* pairs of networks = 19,110.

**Parametric coefficients**	**Estimate**	**t**	**P**
Intercept	0.997	2966.167	**0.001**
Ecoregion (same)	-0.070	-36.419	**0.001**
Biome (same)	-0.002	-3.311	**0.032**
**Smooth Terms**	**EDF**	**F**	**P**
s (human disturbance distance)	8.536	30.036	**0.001**
s (spatial distance)	8.785	65.100	**0.001**
s (elevational difference)	6.187	47.586	**0.001**
s (hours distance)	1.532	5.585	0.299
s (months distance)	5.511	6.974	0.076
s (years distance)	7.217	11.890	**0.019**
s (sampling intensity distance)	1.085	4.382	0.377
s (methods distance)	7.849	15.996	**0.004**

Bold values indicate statistically significant results ($P < 0.05$).

Supplementary Table 23. Multiple predictors of plant-frugivore interaction dissimilarity (β_{WN}). Here, we used a buffer zone of 1000 km and the alternative scenario 2 (see Alternative scenarios section) during the data cleaning process. The binary versions of ecoregion and biome distance matrices were used for estimating the effects of ecoregion and biome borders on the response variable. *P* values were calculated using a two-tailed statistical test that combines Generalized Additive Models (GAM) and Multiple Regression on distance Matrices (MRM). In this approach, the non-independence of distances from each local network is accounted for in the hypothesis testing by performing 1,000 permutations of the response matrix (see Methods). EDF represents the estimated degrees of freedom for each smooth term in the model. *N* pairs of networks = 19,110.

**Parametric coefficients**	**Estimate**	**t**	**P**
Intercept	0.997	3002.382	**0.001**
Ecoregion (same)	-0.069	-36.474	**0.001**
Biome (same)	-0.002	-3.312	**0.048**
**Smooth Terms**	**EDF**	**F**	**P**
s (human disturbance distance)	8.551	30.506	**0.002**
s (spatial distance)	8.782	64.153	**0.001**
s (elevational difference)	6.109	47.538	**0.001**
s (hours distance)	1.579	5.376	0.311
s (months distance)	5.483	7.037	0.054
s (years distance)	7.217	11.954	**0.017**
s (sampling intensity distance)	1.003	5.036	0.320
s (methods distance)	7.867	16.089	**0.004**

Bold values indicate statistically significant results ($P < 0.05$).

Supplementary Table 24. **Multiple predictors of plant-frugivore interaction dissimilarity (β_{WN}).** Here, we used a buffer zone of 1000 km and the alternative scenario 3 (see Alternative scenarios section) during the data cleaning process. The binary versions of ecoregion and biome distance matrices were used for estimating the effects of ecoregion and biome borders on the response variable. *P* values were calculated using a two-tailed statistical test that combines Generalized Additive Models (GAM) and Multiple Regression on distance Matrices (MRM). In this approach, the non-independence of distances from each local network is accounted for in the hypothesis testing by performing 1,000 permutations of the response matrix (see Methods). EDF represents the estimated degrees of freedom for each smooth term in the model. *N* pairs of networks = 19,110.

**Parametric coefficients**	**Estimate**	**t**	**P**
Intercept	0.997	2964.095	**0.001**
Ecoregion (same)	-0.070	-36.404	**0.001**
Biome (same)	-0.002	-3.318	**0.042**
**Smooth Terms**	**EDF**	**F**	**P**
s (human disturbance distance)	8.534	29.989	**0.002**
s (spatial distance)	8.785	65.276	**0.001**
s (elevational difference)	6.170	47.687	**0.001**
s (hours distance)	1.547	5.482	0.300
s (months distance)	5.491	6.909	0.073
s (years distance)	7.210	11.857	**0.020**
s (sampling intensity distance)	1.026	4.983	0.287
s (methods distance)	7.849	16.010	**0.003**

Bold values indicate statistically significant results (*P* < 0.05).

Supplementary Table 25. **Multiple predictors of plant-frugivore network structural dissimilarity.** Here, we used a buffer zone of 500 km and the alternative scenario 1 (see Alternative scenarios section) during the data cleaning process. The binary versions of ecoregion and biome distance matrices were used for estimating the effects of ecoregion and biome borders on the response variable. *P* values were calculated using a two-tailed statistical test that combines Generalized Additive Models (GAM) and Multiple Regression on distance Matrices (MRM). In this approach, the non-independence of distances from each local network is accounted for in the hypothesis testing by performing 1,000 permutations of the response matrix (see Methods). EDF represents the estimated degrees of freedom for each smooth term in the model. *N* pairs of networks = 19,110.

**Parametric coefficients**	**Estimate**	**t**	**P**
Intercept	2.686	221.962	**0.004**
Ecoregion (same)	0.044	0.646	0.775
Biome (same)	-0.024	-1.115	0.826
**Smooth Terms**	**EDF**	**F**	**P**
s (human disturbance distance)	5.948	9.481	0.439
s (spatial distance)	8.473	20.322	**0.015**
s (elevational difference)	8.233	5.501	0.724
s (hours distance)	8.051	7.960	0.968
s (months distance)	6.239	7.217	0.667
s (years distance)	6.830	13.941	0.497
s (sampling intensity distance)	8.759	240.837	**0.001**
s (methods distance)	8.595	17.496	0.233

Bold values indicate statistically significant results (*P* < 0.05).

Supplementary Table 26. Multiple predictors of plant-frugivore network structural dissimilarity. Here, we used a buffer zone of 500 km and the alternative scenario 2 (see Alternative scenarios section) during the data cleaning process. The binary versions of ecoregion and biome distance matrices were used for estimating the effects of ecoregion and biome borders on the response variable. *P* values were calculated using a two-tailed statistical test that combines Generalized Additive Models (GAM) and Multiple Regression on distance Matrices (MRM). In this approach, the non-independence of distances from each local network is accounted for in the hypothesis testing by performing 1,000 permutations of the response matrix (see Methods). EDF represents the estimated degrees of freedom for each smooth term in the model. *N* pairs of networks = 19,110.

**Parametric coefficients**	**Estimate**	**t**	**P**
Intercept	2.685	222.539	**0.002**
Ecoregion (same)	0.084	1.229	0.561
Biome (same)	-0.024	-1.157	0.801
**Smooth Terms**	**EDF**	**F**	**P**
s (human disturbance distance)	5.417	9.472	0.460
s (spatial distance)	8.587	28.061	**0.002**
s (elevational difference)	7.800	3.418	0.904
s (hours distance)	8.088	7.568	0.973
s (months distance)	7.129	7.330	0.682
s (years distance)	6.823	12.437	0.555
s (sampling intensity distance)	8.758	275.291	**0.001**
s (methods distance)	8.550	18.139	0.191

Bold values indicate statistically significant results ($P < 0.05$).

Supplementary Table 27. Multiple predictors of plant-frugivore network structural dissimilarity. Here, we used a buffer zone of 100 km and the alternative scenario 1 (see Alternative scenarios section) during the data cleaning process. The binary versions of ecoregion and biome distance matrices were used for estimating the effects of ecoregion and biome borders on the response variable. *P* values were calculated using a two-tailed statistical test that combines Generalized Additive Models (GAM) and Multiple Regression on distance Matrices (MRM). In this approach, the non-independence of distances from each local network is accounted for in the hypothesis testing by performing 1,000 permutations of the response matrix (see Methods). EDF represents the estimated degrees of freedom for each smooth term in the model. *N* pairs of networks = 19,110.

**Parametric coefficients**	**Estimate**	**t**	**P**
Intercept	2.691	222.709	**0.007**
Ecoregion (same)	0.052	0.757	0.743
Biome (same)	-0.028	-1.364	0.762
**Smooth Terms**	**EDF**	**F**	**P**
s (human disturbance distance)	5.834	9.562	0.428
s (spatial distance)	8.470	20.654	**0.018**
s (elevational difference)	8.080	4.412	0.817
s (hours distance)	8.130	8.456	0.965
s (months distance)	6.321	7.283	0.647
s (years distance)	6.827	13.789	0.501
s (sampling intensity distance)	8.745	241.194	**0.003**
s (methods distance)	8.590	17.524	0.209

Bold values indicate statistically significant results ($P < 0.05$).

Supplementary Table 28. **Multiple predictors of plant-frugivore network structural dissimilarity.** Here, we used a buffer zone of 100 km and the alternative scenario 2 (see Alternative scenarios section) during the data cleaning process. The binary versions of ecoregion and biome distance matrices were used for estimating the effects of ecoregion and biome borders on the response variable. *P* values were calculated using a two-tailed statistical test that combines Generalized Additive Models (GAM) and Multiple Regression on distance Matrices (MRM). In this approach, the non-independence of distances from each local network is accounted for in the hypothesis testing by performing 1,000 permutations of the response matrix (see Methods). EDF represents the estimated degrees of freedom for each smooth term in the model. *N* pairs of networks = 19,110.

**Parametric coefficients**	**Estimate**	**t**	**P**
Intercept	2.684	222.432	**0.004**
Ecoregion (same)	0.089	1.311	0.549
Biome (same)	-0.023	-1.085	0.812
**Smooth Terms**	**EDF**	**F**	**P**
s (human disturbance distance)	5.330	9.475	0.436
s (spatial distance)	8.590	28.764	**0.003**
s (elevational difference)	1.026	4.544	0.803
s (hours distance)	8.122	7.758	0.981
s (months distance)	7.189	7.442	0.677
s (years distance)	6.821	12.365	0.583
s (sampling intensity distance)	8.761	275.772	**0.001**
s (methods distance)	8.540	17.893	0.205

Bold values indicate statistically significant results ($P < 0.05$).

Supplementary Table 29. **Multiple predictors of plant-frugivore network structural dissimilarity.** Here, we used a buffer zone of 100 km and the alternative scenario 3 (see Alternative scenarios section) during the data cleaning process. The binary versions of ecoregion and biome distance matrices were used for estimating the effects of ecoregion and biome borders on the response variable. *P* values were calculated using a two-tailed statistical test that combines Generalized Additive Models (GAM) and Multiple Regression on distance Matrices (MRM). In this approach, the non-independence of distances from each local network is accounted for in the hypothesis testing by performing 1,000 permutations of the response matrix (see Methods). EDF represents the estimated degrees of freedom for each smooth term in the model. *N* pairs of networks = 19,110.

**Parametric coefficients**	**Estimate**	**t**	**P**
Intercept	2.689	222.557	**0.008**
Ecoregion (same)	0.044	0.639	0.754
Biome (same)	-0.031	-1.443	0.741
**Smooth Terms**	**EDF**	**F**	**P**
s (human disturbance distance)	5.869	9.131	0.446
s (spatial distance)	8.479	20.589	**0.021**
s (elevational difference)	8.217	5.476	0.755
s (hours distance)	8.052	7.939	0.966
s (months distance)	6.005	7.020	0.675
s (years distance)	6.834	14.956	0.411
s (sampling intensity distance)	8.746	238.220	**0.003**
s (methods distance)	8.583	17.496	0.206

Bold values indicate statistically significant results ($P < 0.05$).

Supplementary Table 30. **Multiple predictors of plant-frugivore network structural dissimilarity.** Here, we used a buffer zone of 1000 km and the alternative scenario 1 (see Alternative scenarios section) during the data cleaning process. The binary versions of ecoregion and biome distance matrices were used for estimating the effects of ecoregion and biome borders on the response variable. *P* values were calculated using a two-tailed statistical test that combines Generalized Additive Models (GAM) and Multiple Regression on distance Matrices (MRM). In this approach, the non-independence of distances from each local network is accounted for in the hypothesis testing by performing 1,000 permutations of the response matrix (see Methods). EDF represents the estimated degrees of freedom for each smooth term in the model. *N* pairs of networks = 19,110.

**Parametric coefficients**	**Estimate**	**t**	**P**
Intercept	2.687	222.335	**0.001**
Ecoregion (same)	0.047	0.681	0.776
Biome (same)	-0.026	-1.251	0.802
**Smooth Terms**	**EDF**	**F**	**P**
s (human disturbance distance)	5.954	9.761	0.432
s (spatial distance)	8.483	20.514	**0.010**
s (elevational difference)	8.243	5.492	0.736
s (hours distance)	8.009	7.896	0.970
s (months distance)	6.128	6.943	0.699
s (years distance)	6.852	13.832	0.496
s (sampling intensity distance)	8.789	245.694	**0.002**
s (methods distance)	8.593	17.437	0.229

Bold values indicate statistically significant results ($P < 0.05$).

Supplementary Table 31. **Multiple predictors of plant-frugivore network structural dissimilarity.** Here, we used a buffer zone of 1000 km and the alternative scenario 2 (see Alternative scenarios section) during the data cleaning process. The binary versions of ecoregion and biome distance matrices were used for estimating the effects of ecoregion and biome borders on the response variable. *P* values were calculated using a two-tailed statistical test that combines Generalized Additive Models (GAM) and Multiple Regression on distance Matrices (MRM). In this approach, the non-independence of distances from each local network is accounted for in the hypothesis testing by performing 1,000 permutations of the response matrix (see Methods). EDF represents the estimated degrees of freedom for each smooth term in the model. *N* pairs of networks = 19,110.

**Parametric coefficients**	**Estimate**	**t**	**P**
Intercept	2.685	222.527	**0.004**
Ecoregion (same)	0.084	1.225	0.562
Biome (same)	-0.022	-1.058	0.844
**Smooth Terms**	**EDF**	**F**	**P**
s (human disturbance distance)	5.417	9.454	0.427
s (spatial distance)	8.588	28.139	**0.008**
s (elevational difference)	7.796	3.409	0.893
s (hours distance)	8.098	7.547	0.977
s (months distance)	7.123	7.341	0.669
s (years distance)	6.851	12.533	0.570
s (sampling intensity distance)	8.757	275.296	**0.001**
s (methods distance)	8.551	18.041	0.182

Bold values indicate statistically significant results (*P* < 0.05).

Supplementary Table 32. Multiple predictors of plant-frugivore network structural dissimilarity. Here, we used a buffer zone of 1000 km and the alternative scenario 3 (see Alternative scenarios section) during the data cleaning process. The binary versions of ecoregion and biome distance matrices were used for estimating the effects of ecoregion and biome borders on the response variable. *P* values were calculated using a two-tailed statistical test that combines Generalized Additive Models (GAM) and Multiple Regression on distance Matrices (MRM). In this approach, the non-independence of distances from each local network is accounted for in the hypothesis testing by performing 1,000 permutations of the response matrix (see Methods). EDF represents the estimated degrees of freedom for each smooth term in the model. *N* pairs of networks = 19,110.

**Parametric coefficients**	**Estimate**	**t**	**P**
Intercept	2.692	223.088	**0.008**
Ecoregion (same)	0.045	0.663	0.766
Biome (same)	-0.033	-1.581	0.748
**Smooth Terms**	**EDF**	**F**	**P**
s (human disturbance distance)	5.943	9.649	0.423
s (spatial distance)	8.491	20.649	**0.013**
s (elevational difference)	8.230	5.556	0.727
s (hours distance)	8.063	8.161	0.956
s (months distance)	5.980	6.955	0.711
s (years distance)	6.778	14.670	0.479
s (sampling intensity distance)	8.792	243.787	**0.001**
s (methods distance)	8.578	17.155	0.237

Bold values indicate statistically significant results ($P < 0.05$).

Supplementary Table 33. Multiple predictors of plant-frugivore interaction dissimilarity (β_{WN}). Here, we used the binary versions of ecoregion and biome distance matrices and removed the study with the greatest number of networks in our dataset (study ID 76)[20] from the data. *P* values were calculated using a two-tailed statistical test that combines Generalized Additive Models (GAM) and Multiple Regression on distance Matrices (MRM). In this approach, the non-independence of distances from each local network is accounted for in the hypothesis testing by performing 1,000 permutations of the response matrix (see Methods). EDF represents the estimated degrees of freedom for each smooth term in the model. *N* pairs of networks = 12,880.

**Parametric coefficients**	**Estimate**	**t**	**P**
Intercept	0.995	2816.925	**0.001**
Ecoregion (same)	-0.077	-33.132	**0.001**
Biome (same)	-0.0008	-1.254	0.380
**Smooth Terms**	**EDF**	**F**	**P**
s (human disturbance distance)	6.871	11.919	**0.005**
s (spatial distance)	8.917	139.693	**0.001**
s (elevational difference)	5.502	9.025	**0.035**
s (hours distance)	2.007	7.295	0.106
s (months distance)	7.806	23.758	**0.001**
s (years distance)	8.500	33.731	**0.001**
s (sampling intensity distance)	1.002	0.015	0.992
s (methods distance)	8.571	61.413	**0.001**

Bold values indicate statistically significant results ($P < 0.05$).

Supplementary Table 34. Multiple predictors of plant-frugivore network structural dissimilarity. Here, we used the binary versions of ecoregion and biome distance matrices and removed the study with the greatest number of networks in our dataset (study ID 76)[20] from the data. *P* values were calculated using a two-tailed statistical test that combines Generalized Additive Models (GAM) and Multiple Regression on distance Matrices (MRM). In this approach, the non-independence of distances from each local network is accounted for in the hypothesis testing by performing 1,000 permutations of the response matrix (see Methods). EDF represents the estimated degrees of freedom for each smooth term in the model. *N* pairs of networks = 12,880.

**Parametric coefficients**	**Estimate**	**t**	**P**
Intercept	2.568	184.419	**0.022**
Ecoregion (same)	-0.075	-0.826	0.544
Biome (same)	0.041	1.595	0.679
**Smooth Terms**	**EDF**	**F**	**P**
s (human disturbance distance)	4.419	13.240	0.121
s (spatial distance)	8.540	27.067	**0.005**
s (elevational difference)	7.486	11.064	0.364
s (hours distance)	7.717	8.123	0.923
s (months distance)	6.900	5.378	0.714
s (years distance)	5.505	10.312	0.424
s (sampling intensity distance)	8.534	126.502	**0.008**
s (methods distance)	8.489	14.492	0.190

Bold values indicate statistically significant results ($P < 0.05$).

Supplementary Table 35. Multiple predictors of interaction rewiring (β_{OS}) on plant-frugivore networks. Here, we show the results from a Generalized Additive Mixed-effects Model (GAMM) using network IDs as random effects (one random factor for each of the pairs across which distance is compared) to account for the non-independence of distances (see Rewiring analysis section). *P* values of smooth terms are associated with Wald-type tests of smooth components' equality to zero. Linear terms are used for the categorical variables (ecoregions and biomes). EDF represents the estimated degrees of freedom for each smooth term in the model. *N* pairs of networks = 1,314.

**Parametric coefficients**	**Estimate**	**t**	**P**
Intercept	0.576	25.267	**$2x10^{-16}$**
Ecoregion (same)	-0.017	-0.643	0.521
Biome (same)	-0.033	-1.354	0.176
**Smooth Terms**	**EDF**	**F**	**P**
s (human disturbance distance)	1.864	5.039	**0.005**
s (spatial distance)	2.861	17.983	**$2x10^{-16}$**
s (elevational difference)	3.114	13.422	**$2x10^{-16}$**
s (hours distance)	1.000	5.634	**0.018**
s (months distance)	3.434	1.441	0.139
s (years distance)	1.000	0.906	0.341
s (sampling intensity distance)	2.026	3.767	**0.023**
s (methods distance)	1.000	5.129	**0.024**

Bold values indicate statistically significant results ($P < 0.05$).

Supplementary Table 36. The effect of large-scale ecological boundaries on interaction rewiring (β_{OS}). Here, we show the results from a Generalized Additive Mixed-effects Model (GAMM) using ecoregion and biome distance metrics as predictors and network IDs as random effects (one random factor for each of the pairs across which distance is compared) to account for the non-independence of distances (see Rewiring analysis section). Note that, contrary to the full model (Supplementary Table 35), only categorical variables are included in this model (with a fixed effect for each level of the category). The effect of ecoregion boundaries is significant, likely because of their collinearity with our other predictor variables. *N* pairs of networks = 1,314.

Parametric coefficients	Estimate	t	P
Intercept	0.579	22.975	**$2x10^{-16}$**
Ecoregion (same)	-0.155	-6.756	**$2.13x10^{-11}$**
Biome (same)	0.005	0.202	0.84

Bold values indicate statistically significant results ($P < 0.05$).

Supplementary Note

Prisma flowchart

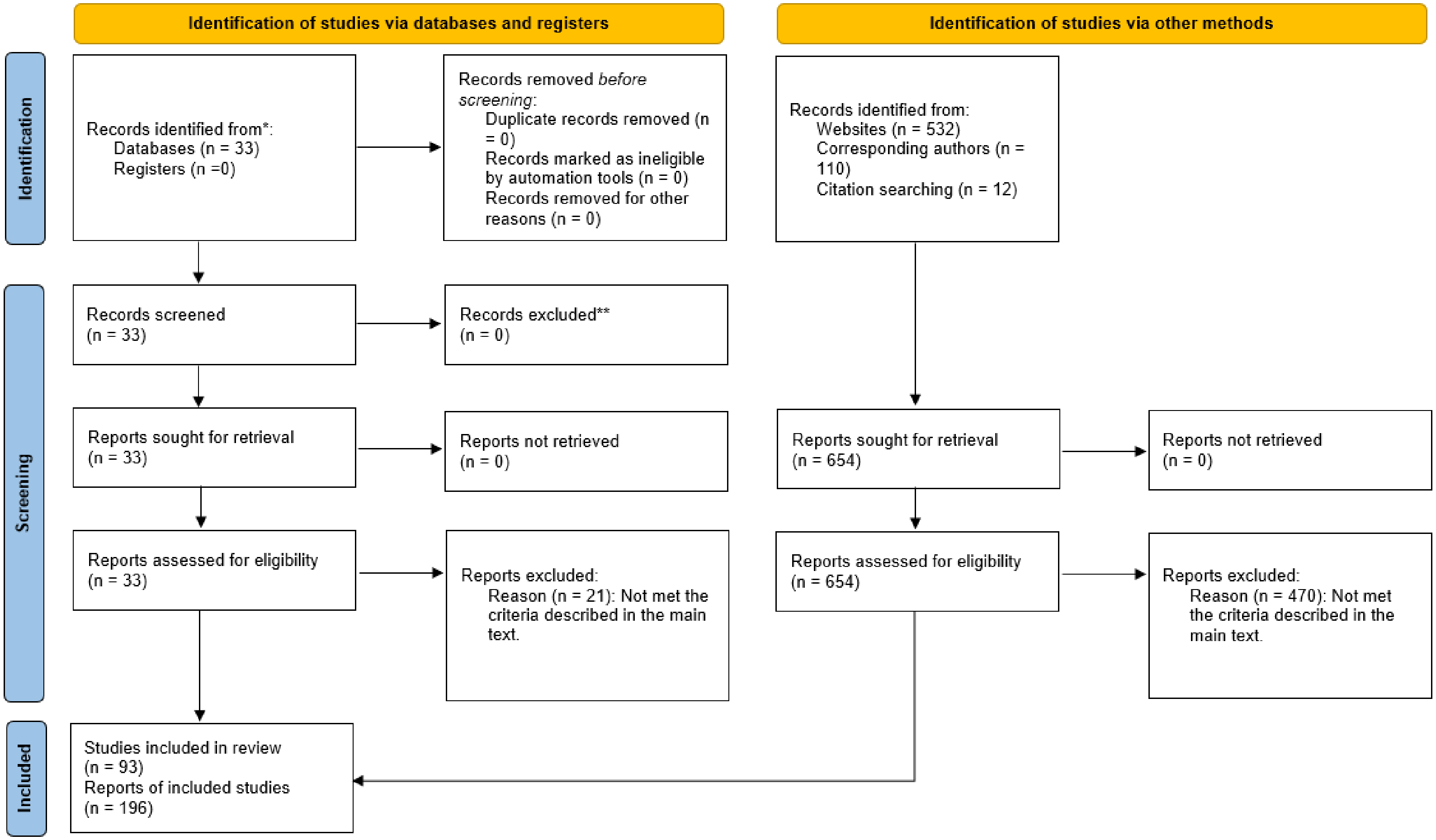

Supplementary References

1. Global Names Resolver: Global Names resolution tools and services. https://resolver.globalnames.org. (2020).
2. Chamberlain, S. A. & Szöcs, E. taxize: taxonomic search and retrieval in R. *F1000Research* **2**, 191 (2013).
3. Rees, T. Taxamatch, an algorithm for near ('Fuzzy’) matching of scientific names in taxonomic databases. *PLoS One* **9**, e107510 (2014).
4. National Center for Biotechnology Information. https://www.ncbi.nlm.nih.gov./ (2020).
5. BirdLife International. https://www.birdlife.org/ (2020).
6. Integrated Taxonomic Information System. https://itis.gov/ (2020).
7. Handbook of the birds of the world and BirdLife International digital checklist of the birds of the world. Version 4.0. http://datazone.birdlife.org/species/taxonomy (2019).
8. Avibase, The world bird database. https://avibase.bsc-eoc.org/ (2020).
9. R Core Team. R: A language and environment for statistical computing (R Foundation for Statistical Computing, Vienna, Austria, 2020).
10. International Plant Name Index. https://www.ipni.org/ (2020).
11. Tropicos: connecting the world to botanical data since 1982. http://www.tropicos.org/ (2020).
12. Boyle, B. *et al*. The taxonomic name resolution service: an online tool for automated standardization of plant names. *BMC Bioinformatics* **14**, 16 (2013).
13. GBIF: The Global Biodiversity Information Facility. What is GBIF? https://www.gbif.org/what-is-gbif (2020).
14. Chamberlain *et al*. rgbif: interface to the Global Biodiversity Information Facility API. R

package version 1.4.0 https://cran.r-project.org/package=rgbif (2021).

15. Cagua, E. F., Lustig, A. & Tylianakis, J. M. Environment affects specialisation of plants and pollinators. Preprint at https://www.biorxiv.org/content/10.1101/866772v1 (2019).

16. Gaiji, S. *et al*. Content assessment of the primary biodiversity data published through GBIF network: status, challenges and potentials. *Biodivers. Informatics* **8**, 94–172 (2013).

17. Zizka, A. *et al*. CoordinateCleaner: standardized cleaning of occurrence records from biological collection databases. *Methods Ecol. Evol.* **10**, 744–751 (2019).

18. Natural Earth. http://www.naturalearthdata.com/ (2020)

19. Dinerstein, E. *et al.* An ecoregion-based approach to protecting half the terrestrial realm. *Bioscience* **67**, 534–545 (2017).

20. Vollstädt, M. G. R. *et al*. Seed-dispersal networks respond differently to resource effects in open and forest habitats. *Oikos* **127**, 847–854 (2017).

21. Baird, J. W. The selection and use of fruit by birds in an eastern forest. *Wilson Bull.* **92**, 63–73 (1980).

22. Carlo, T. A., Collazo, J. A. & Groom, M. J. Avian fruit preferences across a Puerto Rican forested landscape: pattern consistency and implications for seed removal. *Oecologia* **134**, 119–131 (2003).

23. Frost, P. G. H. Fruit-frugivore interactions in a South African coastal dune forest. *Acta XVII Congressus Internationalis Ornithologici* **2**, 1179–1184 (1980).

24. Galetti, M. & Pizo, M. A. Fruit eating by birds in a forest fragment in southeastern Brazil. Ararajuba **4**, 71–79 (1996).

25. Kantak, G. E. Observations on some fruit-eating birds in Mexico. *Auk* **96**, 183–186 (1979).

26. Sorensen, A. E. Interactions between birds and fruit in a temperate woodland. *Oecologia* **50**, 242–249 (1981).

27. W. R. Silva, *Patterns of Fruit-frugivores Interactions in two Atlantic Forest Bird Communities of South-eastern Brazil: Implications for Conservation* in *Seed Dispersal and Frugivory: Ecology, Evolution, and Conservation* (eds. Levey, D. J., Silva, W. R., Galetti, M.) 423–435 (CAB International, 2002).

28. Rodrigues, S. L. M. *Rede de Interações entre aves frugívoras e plantas em uma área de Mata Atlântica no Sudeste do Brasil*, thesis, Universidade Federal de São Carlos, Sorocaba, SP (2015).

29. Castro, E. R. *Fenologia reprodutiva do palmito Euterpe edulis (Arecaceae) e sua influência na abundância de aves frugívoras na floresta Atlântica*, thesis, UNESP, Rio Claro, SP (2007).

30. Correia, J. M. S. *Utilização de espécies frutíferas da Mata Atlântica na alimentação da avifauna da Reserva Biológica de Poços das Antas*, thesis, UNDB, Brasília, DF (1997).

31. Alves, K. J. F. *Composição da avifauna e frugivoria por aves em um mosaico sucessional na Mata Atlântica*, thesis, UNESP, Rio Claro, SP (2008).

32. Fadini, R. F.& de Marco Jr, P. Interações entre aves frugívoras e plantas em um fragmento de mata atlântica de Minas Gerais. *Ararajuba* **12**, 97–103 (2004).

33. Kindel, A. *Interações entre plantas ornitocóricas e aves frugívoras na Estação Ecológica de Aracuri, Muitos Capões, RS*, thesis, UFRGS, Porto Alegre, RS (1996).

34. Pizo, M. A. Frugivory and habitat use by fruit-eating birds in a fragmented landscape of southeast Brazil. *Ornitol. Neotrop*. **15**, 117–126 (2004).

35. Athiê, S. *Composição da avifauna e frugivoria por aves em um mosaico de vegetação*

secundária em Rio Claro, região centro-leste do estado de São Paulo, thesis, UFSCAR, São Carlos, SP (2009).

36. Ribeiro da Silva, F. *et al*. The restoration of tropical seed dispersal networks. *Restor. Ecol.* **23**, 852–860 (2015).

37. Hasui, E. *O papel das aves frugívoras na dispersão de sementes em um fragmento de floresta semidecídua secundária em São Paulo*, thesis, USP, São Paulo, SP (1994).

38. Robinson, V. *Índice de importância de diferentes espécies de plantas na atração de aves para uma área reflorestada em Piracicaba*, thesis, UNESP, Rio Claro, SP (2015).

39. Silva, R. F. M. *Interações entre plantas e aves frugívoras no campus da Universidade Federal do Rio de Janeiro*, thesis, UFRJ, Rio de Janeiro, RJ (2011).

40. Heleno, R. H., Ramos, J. A. & Memmott, J. Integration of exotic seeds into an Azorean seed dispersal network. *Biol. Invasions* **15**, 1143–1154 (2013).

41. Schleuning, M., Blüthgen, N., Flörchinger, M., Braun, J., Schaefer, M. H. & Böhning-gaese, K. Specialization and interaction strength in a tropical plant-frugivore network differ among forest strata. *Ecology* **92**, 26–36 (2011).

42. Schneiberg, I. *et al*. Urbanization homogenizes the interactions of plant-frugivore bird networks. *Urban Ecosyst.* **23**, 457–470 (2020).

43. Machado-de-Souza, T., Campos, R. P., Devoto, M. & Varassin, I. G. Local predictors of the structure of a tropical bird-seed dispersal network. *Oecologia* **189**, 421–433 (2019).

44. González-Castro, A., Traveset, A. & Nogales, M. Seed dispersal interactions in the Mediterranean Region: contrasting patterns between islands and mainland. *J. Biogeogr.* **39**, 1938–1947 (2012).

45. Faustino, T. C. & Machado, C. G. Frugivoria por aves em uma área de campo rupestre na

Chapada Diamantina, BA. *Rev. Bras. Ornitol.* **14**, 137–143 (2006).

46. Jordano, P. El ciclo anual de los paseriformes frugívoros en el matorral mediterráneo del sur de España: importancia de su invernada y variaciones interanuales. *Ardeola* **32**, 69–94 (1985).

47. Saavedra, F. *et al.* Functional importance of avian seed dispersers changes in response to human-induced forest edges in tropical seed-dispersal networks. *Oecologia* **176**, 837–848 (2014).

48. Noma, N. & Yumoto, T. Fruiting phenology of animal-dispersed plants in response to winter migration of frugivores in a warm temperate forest on Yakushima Island, Japan. *Ecol. Res.* **12**, 119–129 (1997).

49. Silva, G. B. M. & Pedroni, F. Frugivoria por aves em área de cerrado no município de Uberlândia, Minas Gerais. *Rev. Árvore* **38**, 433–442 (2014).

50. Vizentin-Bugoni, J. *et al.* Structure, spatial dynamics, and stability of novel seed dispersal mutualistic networks in Hawaiʻi. *Science* **364**, 78–82 (2019).

51. Acosta-Rojas, D. C., Jiménez-Franco, M. V., Zapata-Pérez, V. M., De La Rúa, P. & Martínez-López, V. An integrative approach to discern the seed dispersal role of frugivorous guilds in a Mediterranean semiarid priority habitat. *PeerJ* **7**, e7609 (2019).

52. Naniwadekar, R., Chaplod, S., Datta, A., Rathore, A. & Sridhar, H. Large frugivores matter: insights from network and seed dispersal effectiveness approaches. *J. Anim. Ecol.* **88**, 1250–1262 (2019).

53. Montoya-Arango, S., Acevedo-Quintero, J. F. & Parra, J. L. Abundance and size of birds determine the position of the species in plant-frugivore interaction networks in fragmented forests. *Community Ecol.* **20**, 75–82 (2019).

54. García, D., Donoso, I. & Rodríguez-Pérez, J. Frugivore biodiversity and complementarity in interaction networks enhance landscape-scale seed dispersal function. *Funct. Ecol.* **32**, 2742–2752 (2018).

55. Casas, G., Bastazini, V. A. G., Debastiani, V. J. & Pillar, V. D. Assessing sampling sufficiency of network metrics using bootstrap. *Ecol. Complex.* **36**, 268–275 (2018).

56. Fricke, E. C., Tewksbury, J. J. & Rogers, H. S. Defaunation leads to interaction deficits, not interaction compensation, in an island seed dispersal network. *Glob. Chang. Biol.* **24**, e190–e200 (2018).

57. Rumeu, B. *et al.* Predicting the consequences of disperser extinction: richness matters the most when abundance is low. *Funct. Ecol.* **31**, 1910–1920 (2017).

58. Farwig, N., Schabo, D. G. & Albrecht, J. Trait-associated loss of frugivores in fragmented forest does not affect seed removal rates. *J. Ecol.* **105**, 20–28 (2017).

59. Gorchov, D. L., Cornejo, F., Ascorra, C. F. & Jaramillo, M. Dietary overlap between frugivorous birds and bats in the Peruvian Amazon. *Oikos* **74**, 235–250 (1995).

60. Carlo, T. A. & Morales, J. M. Generalist birds promote tropical forest regeneration and increase plant diversity via rare-biased seed dispersal. *Ecology* **97**, 1819–1831 (2016).

61. Ramos-Robles, M., Andresen, E. & Díaz-Castelazo, C. Temporal changes in the structure of a plant-frugivore network are influenced by bird migration and fruit availability. *PeerJ* **4**, e2048 (2016).

62. Sarmento, R., Alves-Costa, C. P., Ayub, A. & Mello, M. A. R. Partitioning of seed dispersal services between birds and bats in a fragment of the Brazilian Atlantic Forest. *Zoologia* **31**, 245–255 (2014).

63. García, D., Martínez, D., Stouffer, D. B. & Tylianakis, J. M. Exotic birds increase

generalization and compensate for native bird decline in plant-frugivore assemblages. *J. Anim. Ecol.* **83**, 1441–1450 (2014).

64. Cruz, J. C., Ramos, J. A., da Silva, L. P., Tenreiro, P. Q. & Heleno, R. H. Seed dispersal networks in an urban novel ecosystem. *Eur. J. For. Res.* **132**, 887–897 (2013).

65. Burns, K. C. What causes size coupling in fruit–frugivore interaction webs? *Ecology* **94**, 295–300 (2013).

66. Andrade, P. C., Mota, J. V. L. & de Carvalho, A. A. F. Interações mutualísticas entre aves frugívoras e plantas em um fragmento urbano de Mata Atlântica, Salvador, BA. *Rev. Bras. Ornitol.* **19**, 63–73 (2011).

67. Velho, N., Ratnam, J., Srinivasan, U. & Sankaran, M. Shifts in community structure of tropical trees and avian frugivores in forests recovering from past logging. *Biol. Conserv.* **153**, 32–40 (2012).

68. O'Donnell, C. F. & Dilks, P. J. Foods and foraging of forest birds in temperate rainforest, South Westland, New Zealand. *N. Z. J. Ecol.* **18**, 87–107 (1994).

69. Costa, J. M., da Silva, L. P., Ramos, J. A. & Heleno, R. H. Sampling completeness in seed dispersal networks: when enough is enough. *Basic Appl. Ecol.* **17**, 155–164 (2016).

70. Timóteo, S., Correia, M., Rodríguez-Echeverría, S., Freitas, H. & Heleno, R. Multilayer networks reveal the spatial structure of seed-dispersal interactions across the Great Rift landscapes. *Nat. Commun.* **9**, 140 (2018).

71. David, J. P., Murugan, B. S. & Manakadan, R. Frugivory by birds and mammals in Sriharikota Island, southern India. *J. Bombay Nat. Hist. Soc.* **108**, 24–40 (2011).

72. Medeiros e Silva, É. E., Paixão, V. H. F., Torquato, J. L., Lunardi, D. G. & Lunardi, V. de O. Fruiting phenology and consumption of zoochoric fruits by wild vertebrates in a

seasonally dry tropical forest in the Brazilian Caatinga. *Acta Oecologica* **105**, 103553 (2020).

73. Pratt, T. K. & Stiles, E. W. The Influence of fruit size and structure on composition of frugivore assemblages in New Guinea. *Biotropica* **17**, 314–321 (1985).

74. Brown, E. D. & Hopkins, M. J. G. Tests of disperser specificity between frugivorous birds and rainforest fruits in New Guinea. *Emu* **102**, 137–146 (2002).

75. Kopij, G. Winter diet of frugivorous birds in the suburbs of Bloemfontein, South Africa. *African J. Wildl. Res.* **30**, 163–165 (2000).

76. Stanley, M. C. & Lill, A. Avian fruit consumption and seed dispersal in a temperate Australian woodland. *Austral Ecol.* **27**, 137–148 (2002).

77. French, K. Evidence for frugivory by birds in montane and lowland forests in south-east Australia. *Emu* **90**, 185–189 (1990).

78. Williams, P. A. & Karl, B. J. Fleshy fruits of indigenous and adventive plants in the diet of birds in forest remnants, Nelson, New Zealand. *N. Z. J. Ecol.* **20**, 127–145 (1996).

79. Gomes, V. S. M. *Variação espacial e dieta de aves terrestres na restinga de Jurubatiba, RJ*, thesis, UFRJ, Rio de Janeiro, RJ (2006).

80. Motta Jr, J. C. *A exploração de frutos como alimento por aves de mata ciliar numa região do Distrito Federal*, thesis, UNESP, Rio Claro, SP (1991).

81. Argel-de-Oliveira, M. M. *Frugivoria por aves em um fragmento de floresta de restinga no estado do Espírito Santo, Brasil*, thesis, UNICAMP, Campinas, SP (1999).

82. Ikuta, K. G. & Martins, F. C. Interação entre aves frugívoras e plantas no Parque Estadual da Cantareira, estado de São Paulo. *Atualidades Ornitológicas* **172**, 33–36 (2013).

83. Donatti, C. I. *et al.* Analysis of a hyper-diverse seed dispersal network: modularity and

underlying mechanisms. *Ecol. Lett.* **14**, 773–781 (2011).

84. Gondim, M. J. C. *A exploração de frutos por aves frugívoras em uma área de Cerradão no Estado de São Paulo*, thesis, UNESP, Rio Claro, SP (2002).

85. Ruggera, R. A., Blendinger, P. G., Gomez, M. D. & Marshak, C. Linking structure and functionality in mutualistic networks: do core frugivores disperse more seeds than peripheral species? *Oikos* **125**, 541–555 (2016).

86. Blendinger, P. G. *et al.* Scale-dependent spatial match between fruits and fruit-eating birds in Andean mountain forests. *Biotropica* **47**, 702–711 (2015).

87. Blendinger, P. G. *et al.* Fine-tuning the fruit-tracking hypothesis: spatiotemporal links between fruit availability and fruit consumption by birds in Andean mountain forests. *J. Anim. Ecol.* **81**, 1298–1310 (2012).

88. Menezes Pinto, Í., Emer, C., Cazetta, E. & Morante-Filho, J. C. Deforestation simplifies understory bird seed-dispersal networks in human-modified landscapes. *Front. Ecol. Evol.* **9**, 640210 (2021).

89. Boyle, W. A., Conway, C. J. & Bronstein, J. L. Why do some, but not all, tropical birds migrate? A comparative study of diet breadth and fruit preference. *Evol. Ecol.* **25**, 219–236 (2011).

90. Crome, F. H. J. Foraging ecology of an assemblage of birds in lowland rainforest in northern Queensland. *Aust. J. Ecol.* **3**, 195–212 (1978).

91. Gopal, A., Mudappa, D., Raman, T. R. S. & Naniwadekar, R. Forest cover and fruit crop size differentially influence frugivory of select rainforest tree species in Western Ghats, India. *Biotropica* **52**, 871–883 (2020).

92. Li, H. *et al.* The functional roles of species in metacommunities, as revealed by

metanetwork analyses of bird–plant frugivory networks. *Ecol. Lett.* **23**, 1252–1262 (2020).

93. Herrera, C. M. A study of avian frugivores, bird-dispersed plants, and their interaction in Mediterranean scrublands. *Ecol. Monogr.* **54**, 1–23 (1984).

94. Plein, M. *et al.* Constant properties of plant-frugivore networks despite fluctuations in fruit and bird communities in space and time. *Ecology* **94**, 1296–1306 (2013).

95. Stiebel, H. *Frugivorie bei mitteleuropäischen Vögeln*, thesis, Universität Oldenburg (2003).

96. Muñoz, M. C., Schaefer, H. M., Böhning-Gaese, K. & Schleuning, M. Importance of animal and plant traits for fruit removal and seedling recruitment in a tropical forest. *Oikos* **126**, 823–832 (2017).

97. Quitián, M. *et al.* Elevation-dependent effects of forest fragmentation on plant-bird interaction networks in the tropical Andes. *Ecography* **41**, 1497–1506 (2018).

98. Dehling, D. M. *et al.* Functional relationships beyond species richness patterns: trait matching in plant-bird mutualisms across scales. *Glob. Ecol. Biogeogr.* **23**, 1085–1093 (2014).

99. Buitrón-Jurado, G. *Diversidad de aves frugívoras y árboles, redes de interacción e identificación de árboles magnetos en dos bosques nublados de Venezuela con distintas condiciones de fragmentación*, thesis, Instituto Venezolano de Investigaciones Científicas, Miranda, Venezuela (2012).

100. Balasubramanian, P. Interactions between fruit-eating birds and bird-dispersed plants in the tropical dry evergreen forest of Point Calimere, South India. *J. Bombay Nat. Hist. Soc.* **93**, 428–441 (1996).

101. Chimera, C. G. & Drake, D. R. Patterns of seed dispersal and dispersal failure in a

hawaiian dry forest having only introduced birds. *Biotropica* **42**, 493–502 (2010).

102. Githiru, M., Lens, L., Bennur, L. A. & Ogol, C. P. K. O. Effects of site and fruit size on the composition of avian frugivore assemblages in a fragmented Afrotropical forest. *Oikos* **96**, 320–330 (2002).

103. Malmborg, P. K. & Willson, M. F. Foraging ecology of avian frugivores and some consequences for seed dispersal in an Illinois woodlot. *Condor* **90**, 173–186 (1988).

104. Spotswood, E. N., Meyer, J. Y. & Bartolome, J. W. An invasive tree alters the structure of seed dispersal networks between birds and plants in French Polynesia. *J. Biogeogr.* **39**, 2007–2020 (2012).

105. Wolfe, J. D., Johnson, M. D. & Ralph, C. J. Do birds select habitat or food resources? Nearctic-neotropic migrants in northeastern Costa Rica. *PLoS One* **9**, e86221 (2014).

106. Chen, C. C. & Chou, L. S. The diet of forest birds at Fushan Experimental Forest. *Taiwan J. For. Sci.* **14**, 275–287 (1999).

107. Kamruzzaman, M. & Asmat, G. S. M. Seasonal variations of fruit preference among frugivorous birds in Chittagong, Bangladesh. *Bangladesh J. Zool.* **36**, 187–206 (2008).

108. Palmeirim, J. M., Gorchov, D. L. & Stoleson, S. Trophic structure of a neotropical frugivore community: is there competition between birds and bats? *Oecologia* **79**, 403–411 (1989).

109. Blake, J. G. & Loiselle, B. A. Fruits in the diets of neotropical migrant birds in Costa Rica. *Biotropica* **24**, 200–210 (1992).

110. Traveset, A. Resultats preliminars sobre el consum de fruits per ocells a l'illa de Cabrera (Illes Balears). *Anu. Ornitològic les Balear* **7**, 3–9 (1992).

111. Fortuna, M. A., Ortega, R. & Bascompte, J. The web of life. Preprint at

https://arxiv.org/abs/1403.2575 (2014).

112. Schleuning, M. *et al.* Specialization of mutualistic interaction networks decreases toward tropical latitudes. *Curr. Biol.* **22**, 1925–1931 (2012).

113. Dalsgaard, B. *et al.* Opposed latitudinal patterns of network-derived and dietary specialization in avian plant–frugivore interaction systems. *Ecography* **40**, 1395–1401 (2017).

114. Chao, A., Colwell, R. K., Lin, C. & Gotelli, N. J. Sufficient sampling for asymptotic minimum species richness estimators. *Ecology* **90**, 1125–1133 (2009).
