# Supplementary material for: Global and regional ecological boundaries explain abrupt spatial discontinuities in avian frugivory interactions
Source: Nat Commun. 2022 Nov 14;13:6943. doi: 10.1038/s41467-022-34355-w (PMC9663448; doi:10.1038/s41467-022-34355-w)
Supplement: Supplementary file 3 — Description to Additional Supplementary Information [file 41467_2022_34355_MOESM3_ESM.pdf]

File Name: Supplementary Data 1
Description: Metadata of the 196 plant-frugivore networks in our dataset.

File Name: Supplementary Data 2
Description: Predictor and response variables used in our analyses.

File Name: Source Data
Description: Data underlying figures 2-6.
