# Supplementary material for: Global and regional ecological boundaries explain abrupt spatial discontinuities in avian frugivory interactions
Source: Nat Commun. 2022 Nov 14;13:6943. doi: 10.1038/s41467-022-34355-w (PMC9663448; doi:10.1038/s41467-022-34355-w)
Supplement: Supplementary file 6 — Reporting Summary [file 41467_2022_34355_MOESM6_ESM.pdf]

nature portfolio

Corresponding author(s): Lucas P. Martins
Jason M. Tylianakis

Last updated by author(s): 17/10/22

Reporting Summary

Nature Portfolio wishes to improve the reproducibility of the work that we publish. This form provides structure for consistency and transparency in reporting. For further information on Nature Portfolio policies, see our Editorial Policies and the Editorial Policy Checklist.

Statistics

For all statistical analyses, confirm that the following items are present in the figure legend, table legend, main text, or Methods section.

n/a	Confirmed	
☐	☒	The exact sample size (*n*) for each experimental group/condition, given as a discrete number and unit of measurement
☐	☒	A statement on whether measurements were taken from distinct samples or whether the same sample was measured repeatedly
☐	☒	The statistical test(s) used AND whether they are one- or two-sided<br>*Only common tests should be described solely by name; describe more complex techniques in the Methods section.*
☐	☒	A description of all covariates tested
☐	☒	A description of any assumptions or corrections, such as tests of normality and adjustment for multiple comparisons
☐	☒	A full description of the statistical parameters including central tendency (e.g. means) or other basic estimates (e.g. regression coefficient) AND variation (e.g. standard deviation) or associated estimates of uncertainty (e.g. confidence intervals)
☐	☒	For null hypothesis testing, the test statistic (e.g. *F*, *t*, *r*) with confidence intervals, effect sizes, degrees of freedom and *P* value noted<br>*Give P values as exact values whenever suitable.*
☒	☐	For Bayesian analysis, information on the choice of priors and Markov chain Monte Carlo settings
☒	☐	For hierarchical and complex designs, identification of the appropriate level for tests and full reporting of outcomes
☐	☒	Estimates of effect sizes (e.g. Cohen's *d*, Pearson's *r*), indicating how they were calculated

Our web collection on statistics for biologists contains articles on many of the points above.

Software and code

Policy information about availability of computer code

Data collection	No software was used for collecting plant-frugivore network data.
Data analysis	We used R version 3.6.1. and packages 'mgcv' version 1.8-31 and 'ecodist' version 2.0.3 to perform our GAM-based MRM models. Generalized Additive Mixed Models were performed using the 'gamm4' package version 0.2-6. Facets of network dissimilarity were calculated using the packages 'betalink' version 2.2.1 (for interaction dissimilarity, species turnover and interaction rewiring) and 'bipartite' version 2.15 (for metrics of network structure). We used the 'raster' package version 3.1-5 to read and analyse climatic and spatial data. The following packages were also used for perfoming analyses or standardizing the taxonomy of bird and plant species: 'vegan' version 2.5-3, 'ade4' version 1.7-15, 'taxize' version 0.9.91, 'rgbif' version 1.4.0 and 'CoordinateCleaner' version 2.0-11.<br>The data and scripts necessary to reproduce our analyses have been deposited in the Dryad database: https://doi.org/10.5061/dryad.mcvdnck4d .

For manuscripts utilizing custom algorithms or software that are central to the research but not yet described in published literature, software must be made available to editors and reviewers. We strongly encourage code deposition in a community repository (e.g. GitHub). See the Nature Portfolio guidelines for submitting code & software for further information.

Data

Policy information about availability of data

All manuscripts must include a data availability statement. This statement should provide the following information, where applicable:

- Accession codes, unique identifiers, or web links for publicly available datasets
- A description of any restrictions on data availability
- For clinical datasets or third party data, please ensure that the statement adheres to our policy

We obtained ecoregions and biome maps from Ecoregions 2017 © Resolve (Dinerstein et al. 2017; see references). This shapefile is available at https://ecoregions.appspot.com/ under a CC-BY 4.0 license. Human footprint data (developed by Venter et al. 2016; see references) are publicly available at https://doi.org/10.5061/dryad.052q5. Climatic and elevational data were obtained from Worldclim 2.1 and are pubicly available at https://www.worldclim.org/. Networks were obtained through different online sources and publications or by contacting corresponding authors (see Supplementary Table 1). Plant-frugivore network data used in our analyses (i.e., after data cleaning) are available at https://doi.org/10.5061/dryad.mcvdnck4d (see Data availability). The following taxonomic databases were used for standardizing the taxonomy of plant and bird species in our dataset: Global Names Resolver (GNR) (available at https://resolver.globalnames.org/), National Center for Biotechnology Information (NCBI) (available at https://ncbi.nlm.nih.gov/), BirdLife International (available at http://datazone.birdlife.org/species/taxonomy), Avibase (available at https://avibase.bsc-eoc.org/), Integrated Taxonomic Information System (ITIS) (available at https://itis.gov/), International Plant Names Index (IPNI) (available at https://www.ipni.org/), Tropicos (available at https://www.tropicos.org/), and the iPlant Taxonomic Name Resolution Service (available at https://tnrs.biendata.org/). Source data are provided with this paper.

Field-specific reporting

Please select the one below that is the best fit for your research. If you are not sure, read the appropriate sections before making your selection.

☐ Life sciences ☐ Behavioural & social sciences ☒ Ecological, evolutionary & environmental sciences

For a reference copy of the document with all sections, see nature.com/documents/nr-reporting-summary-flat.pdf

Ecological, evolutionary & environmental sciences study design

All studies must disclose on these points even when the disclosure is negative.

Study description	The major aim of this study was to test whether large-scale ecological boundaries (ecoregions and biomes) and gradients of human disturbance increase dissimilarity among plant-frugivore networks, while accounting for effects of spatial and elevational gradients and differences in network sampling. To tackle this aim, we assembled a large database comprising 196 quantitative local networks of avian frugivory. We generated the following distance matrices (N × N, where N is the number of local networks in our dataset; N pairs of networks = 19,110) to be variables in the statistical models: ecoregion, biome, human footprint, spatial, elevational, hours, months, years, sampling intensity and methods. Facets of network dissimilarity (interaction dissimilarity, species turnover and network structural dissimilarity) were used as response variables. We then employed a combination of Generalized Additive Models (GAM) and Multiple Regression on distance Matrices (MRM) to evaluate the effect of each of our predictor distance matrices on our response matrix (see Methods). This analysis is equivalent to a GAM, but where the predictor and response variables are distance matrices and the non-independence of distances from each local network is accounted for in the hypothesis testing by permuting the response matrix (N = 1,000 permutations). Because interaction rewiring can only be calculated for networks that share species from both trophic levels, we selected a subset of network pairs that shared plants and birds (N = 1,314) to test whether interaction rewiring increases across large-scale environmental gradients. Importantly, since not all possible combinations of network pairs contained values of interaction rewiring (i.e., not all pairs of networks shared species), a pairwise distance matrix could not be generated for this metric. Thus, we performed a Generalized Additive Mixed Model (GAMM) using ecoregion, biome, spatial, elevational, and sampling-related distance metrics as fixed effects and network IDs as random effects (to account for the non-independence of distances).
Research sample	We chose to test our hypothesis using plant-frugivore networks because of their importance for seed dispersal, promoting species diversity and regenerating degraded ecosystems. We removed non-avian frugivores (e.g., mammals, reptiles, fishes) from our networks to avoid overestimating network dissimilarity due to sampling differences in focal taxa. In addition, birds have well known taxonomy, which contributed to the process of standardizing the taxonomy of the species in our networks (Supplementary Figs. 3-6). In the end, we obtained 196 quantitative avian frugivory networks from 93 different studies. These studies differed in sampling effort and methods, but these variables were controlled for in our models. Because some of these studies contained multiple networks, we evaluated whether our results were strongly biased by individual studies. To do this, we tested whether F-values of smooth terms and t-values of categorical variables (for the binary version of ecoregion and biome distance matrices) changed significantly when jackknifing across studies. We did this by dropping one study from the dataset and re-fitting the models, and then repeating this same process for all the studies in our dataset. We also performed a sensitivity analysis to evaluate the effect of changing the threshold of minimum network size used for analyses on the reported patterns.
Sampling strategy	Network data were obtained from online sources, published literature or by contacting corresponding authors (see Methods). The number of networks that met previously established criteria (see section below) determined the sample size of our analyses (N studies = 93; N networks = 196; N pairs of networks = 19,110). We assessed the robustness of our findings to sampling size by performing two analyses: in the first, we removed individual studies from the dataset and re-fitted our models; in the second, we ran a sensitivity analysis in which networks with low number of species are progressively removed from the dataset. Tests of our key hypotheses were robust to both procedures, indicating that even smaller sampling sizes would be sufficient for our analysis.
Data collection	Plant-frugivore network data were obtained through different online sources and publications (Supplementary Table 1). Only

Data collection	networks that met the following criteria were retrieved: (i) the network contains quantitative data (a measure of interaction frequency) from a location, pooling through time if necessary; (ii) the network includes avian frugivores; (iii) the network (after removal of non-avian frugivores) contains greater than two species in each trophic level. Because this size threshold was somewhat arbitrary, we used a sensitivity analysis to assess the effect of our network size threshold on the reported patterns; and (iv) network sampling was not taxonomically restricted, that is, sampling was not focused on a specific taxonomic group, such as a given plant or bird family. Note, however, that authors often select focal plants or frugivorous birds to be sampled, but this was not considered as a taxonomic restriction if plants and birds were not selected based on their taxonomy (e.g., focal plants were selected based on the availability of fruits at the time of sampling, or focal birds were selected based on previous studies of bird diet in the study site). The first source for network data was the Web of Life database, which contains 33 georeferenced plant-frugivore networks from 28 published studies, of which 12 networks met our criteria.<br>We also accessed the Scopus database on 04 May 2020 using the following keyword combination: (“plant-frugivore*” OR “plant-bird*” OR “frugivorous bird*” OR “avian frugivore*” OR “seed dispers*”) AND (“network*” OR “web*”) to search for papers that include data on avian frugivory networks. The search returned a total of 532 studies, from which 62 networks that met the above criteria were retrieved. We also contacted authors to obtain plant-frugivore networks that were not publicly available, which provided us a further 110 networks. The remaining networks (N =12) were obtained by checking the database from a recently published study (Fricke & Svenning 2020; see references). In total, 196 quantitative avian frugivory networks were used in our analyses. The network dataset was compiled by the first author (Lucas P. Martins).
Timing and spatial scale	Plant-frugivore networks were sampled between 1974 and 2019. Importantly, we included in our models a matrix of differences in sampling year between networks, which aims to account for long-term changes in the environment, species composition and network sampling methods. The coordinates and location of the specific networks can be seen in Supplementary Table 1 and Supplementary Fig. 2. We analysed all the network data that we were able to collate. In the end, our dataset comprised 196 quantitative avian frugivory networks distributed across 67 ecoregions, 11 biomes, and 6 continents.
Data exclusions	We excluded non-avian frugivores from our networks. This pre-established exclusion criterion was defined based on the fact that not removing non-avian frugivores would generate spurious apparent turnover between networks that did vs. did not sample those taxa. We highlight that the removal of non-avian frugivores did not strongly decrease the number of frugivores in our dataset or the total number of links in the global network of frugivory. Furthermore, non-avian frugivores, as well as their interactions, were not shared across ecoregions and biomes, so their inclusion would only strengthen the results we found.
Reproducibility	We used different approaches to validate the generality and reproducibility of our models: i) we tested whether F-values of smooth terms and t-values of categorical variables (for the binary version of ecoregion and biome distance matrices) changed significantly when repeatedly dropping one study from the dataset and re-fitting the models, (ii) we performed a sensitivity analysis to evaluate the effect of changing the threshold of minimum network size used for analyses on the reported patterns; (iii) we measured ecoregion and biome distances using both a binary and a quantitative (based on environmental distance) metrics, and (iv) we performed our models using different buffer zone sizes and scenarios for attributing names for problematic species that could not be considered as unique species in our dataset (see more details in the Supplementary Information).<br><br>We provide the R code necessary to reproduce the analyses of the study (see Code availability).
Randomization	In our GAM-based MRM models, the non-independence of distances from each local network is accounted for in the hypothesis testing by permuting (i.e., shuffling) the response matrix (N = 1,000 permutations). In our Generalized Additive Mixed Model (GAMM), we used network IDs as random effects to account for the non-independence of distances.
Blinding	Although blinding is not possible in our study, we used all the datasets that we were able to collect following pre-established criteria.

Did the study involve field work? ☐ Yes ☒ No

Reporting for specific materials, systems and methods

We require information from authors about some types of materials, experimental systems and methods used in many studies. Here, indicate whether each material, system or method listed is relevant to your study. If you are not sure if a list item applies to your research, read the appropriate section before selecting a response.

Materials & experimental systems

n/a	Involved in the study
☒	☐ Antibodies
☒	☐ Eukaryotic cell lines
☒	☐ Palaeontology and archaeology
☒	☐ Animals and other organisms
☒	☐ Human research participants
☒	☐ Clinical data
☒	☐ Dual use research of concern

Methods

n/a	Involved in the study
☒	☐ ChIP-seq
☒	☐ Flow cytometry
☒	☐ MRI-based neuroimaging
